# Transcriptome Profiling of *Escherichia coli* B During Sequential Adaptation to T4 Phage and Iron(III) Stress

**DOI:** 10.3390/antibiotics15070684

**Published:** 2026-07-13

**Authors:** Franklin C. Ezeanowai, Akamu J. Ewunkem, Danielle Winston, Larisa C. Kiki, Ugonna C. Morikwe, Lindsey W. McGee, Joseph L. Graves, Liesl K. Jeffers-Francis

**Affiliations:** 1 Biology Department, North Carolina Agricultural and Technical State University, 1601 E Market Street, Greensboro, NC 27411, USA; cfezeanowai@aggies.ncat.edu (F.C.E.); dmwinston@aggies.ncat.edu (D.W.); klchila@aggies.ncat.edu (L.C.K.); ucmorikwe@aggies.ncat.edu (U.C.M.); gravesjl@ncat.edu (J.L.G.J.); 2 Department of Biological Sciences, Winston Salem State University, 601 S Martin Luther King Jr Drive, Winston Salem, NC 27110, USA; ewunkemaj@wssu.edu; 3 Department of Biology, Biochemistry, Public Health, Quality Science, Earlham College, 801 National Road West, Richmond, IN 47374, USA; mcgeeli@earlham.edu

**Keywords:** *Escherichia coli* B, RNA-seq, T4 phage, iron(III), differential gene expression, transcriptomics, experimental evolution, antimicrobial resistance, *pmrB*, fitness epistasis

## Abstract

**Background/Objective:** Antimicrobial resistance poses a critical public health crisis, highlighting the urgent requirement to investigate bacterial evolutionary adaptations and pioneer alternative therapeutics. Consequently, bacteriophages and metal-based compounds are emerging as viable options to combat drug-resistant infections. Building on our finding that T4 phage resistance in *E. coli* B also confers adaptation to high iron(III), we used RNA-sequencing (RNA-seq) to explore bacterial gene expression in resistant and control populations. We analyzed samples from our five experimental groups—Ancestor (ANC), control (CON), phage-selected (Phage), iron(III)-selected (FE), and phage/iron(III)-selected (PF), to understand how these regimes drive transcriptional changes. **Method:** Total RNA was extracted using the TRIzol protocol, and sequencing libraries were prepared with the Illumina RNA Total Library Prep Kit. Sequencing was performed on the Illumina NextSeq 1000/2000 platform. Reads were aligned to the *E. coli* B ATCC 11303 reference genome, and pairwise comparisons between the five experimental groups were conducted to determine differential gene expression profiles. **Results:** Principal component analysis (PCA) showed that iron-adapted populations (FE and PF) separated distinctly from the Ancestor and control populations along PC1 (capturing 40% of the variance), while the phage-selected replicates were split, with one (Phage5) clustering with CON3 and two (Phage2, Phage4) falling closer to, but clearly separated from, the Ancestor. Differential expression analysis (P_adj_ < 0.05 and |log_2_FC| ≥ 1) revealed extensive transcriptional rewiring, with 482 and 381 differentially expressed genes (DEGs) in the FE and PF populations, respectively, compared to the Ancestor, and 177 DEGs in the phage-selected population compared to the Ancestor. The direct pairwise comparison between the iron-selected and phage/iron-selected populations yielded zero DEGs, demonstrating that both iron-adapted populations converged on a near-identical gene expression profile regardless of their distinct genetic and evolutionary backgrounds. **Conclusions:** This study suggests that *pmrB* and *arn* pathway genes may serve as primary markers for resistance to iron stress. These results are significant because they demonstrate a coordinated, multi-gene defense mechanism in *E. coli* B against high iron(III) stress, in which the *arn* operon and *eptA* remodel lipid A and the outer membrane, while glycerol-3-phosphate metabolism and phage-shock/chaperone pathways are repressed.

## 1. Introduction

Antimicrobial resistance is increasing worldwide, and there is a need to develop new therapeutics and to understand more about the evolution of bacteria under different selective pressures [1,2]. Bacteriophages (phages) and metal-based compounds are promising alternatives to traditional antibiotics [3,4]. While metal-based substances like iron(III) act through a variety of mechanisms, such as the production of reactive oxygen species, the loss of membrane integrity [5,6], and induction of lipid A modifications that counteract the resulting envelope stress [6,7], phages kill bacteria with a high host specificity [8]. Because these two agents impose fundamentally different selective pressures (one biological and target-specific and the other chemical and broad-acting), they have been proposed for use in combination therapy to limit the emergence of resistance [9].

Although the mechanisms of phage resistance have been extensively studied, there is a growing interest in how these adaptations interact with other environmental stressors, such as heavy metals. For example, iron is a metal that is biologically necessary but can be poisonous to bacteria in higher doses. Iron at optimal levels is essential for energy generation and enzymatic function, but excess iron can cause oxidative stress via Fenton reactions and disrupt key metabolic pathways [10,11,12]. To reduce damage, bacteria exposed to high levels of iron often modify their gene expression profile, activate detoxification pathways, and restructure membranes [6,7,12,13]. These changes may overlap with defense mechanisms to phage infection, raising concerns about how bacteria adapt to both stressors [14,15]. However, the molecular mechanisms underlying bacterial responses to the sequential or combined use of these stressors remain poorly understood, limiting the rational design of effective combination therapies.

Our previous study by Ezeanowai et al. [15] studied the experimental evolution of *Escherichia coli* B ATCC 11303 under four treatment conditions: control (LB broth), phage-selected, iron(III)-selected, and phage/iron(III)-selected for 35 days. Rapid and complete phage resistance developed within 24 h of phage exposure, while the iron(III)-selected population evolved resistance to iron(III) at concentrations up to 1750 mg/L. Genomic analysis from whole-genome sequencing revealed that the phage-selected population carried large deletions in the lipopolysaccharide (LPS) biosynthesis genes (*waaA*, *waaG*), whereas the iron-selected and phage/iron(III)-selected populations carried selective sweeps in iron-associated regulatory and membrane-associated genes (*qseB*, *basR*, *aroK*, *fieF*, *rseB*, *cpxP*) [15]. These contrasting genomic signatures suggest that phage and iron resistance are achieved through fundamentally different routes, which are structural alteration of the cell surface and regulatory rewiring. While genomic data have clarified the mutational changes supporting these adaptations, the transcriptomic consequences of these adaptations remain unknown. Understanding that bacteria frequently change regulatory pathways, which reshape the transcriptome without corresponding coding-sequence mutations, using only genomic data cannot reveal whether mutations translate into altered gene expression [16,17].

In this study, we performed RNA sequencing to characterize the transcriptomic changes associated with phage selection, exposure to elevated iron(III), and their sequential combination in experimentally evolved *Escherichia coli* B populations. We evaluated three hypotheses: (1) phage-selected and iron-selected populations will exhibit different transcriptomic patterns, reflecting the different selection pressures they experienced; (2) sequential phage/iron selection will result in a transcriptomic pattern that is unique to either iron or phage stressor, reflecting the influence of previous adaptation on subsequent transcriptional responses; and (3) differentially expressed genes will map to functional pathways consistent with the genomic mutations previously identified, including LPS biosynthesis, iron homeostasis and membrane-associated regulatory systems. Together, these analyses aimed at identifying functionally relevant pathways, to evaluate transcriptomic convergence or divergence among resistant populations, and to determine how initial adaptation to one stressor modifies the transcriptomic response upon exposure to a second stressor.

## 2. Results

### 2.1. Pair-Wise Comparison of the Experimental Groups

Pairwise comparisons across treatment groups were performed. Differential expression thresholds were set at P_adj_ < 0.05 and |log_2_FC| ≥ 1. Table 1 summarizes DEG counts for all 10 comparisons.

### 2.2. The Principal Component Analysis of All E. coli B Populations

**Figure 1:**
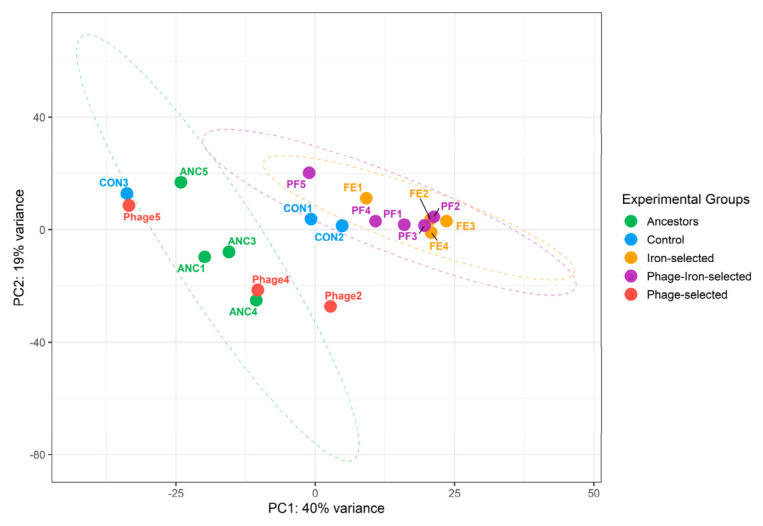
Principal component analysis (PCA) of all 19 *E. coli* B samples. PC1 (40% of the variance) separates the iron-adapted populations (FE, PF) from the Ancestor and control populations; PC2 (19% of the variance) separates the Ancestor replicates ANC1, ANC3, and ANC4 from the FE/PF cluster. The phage-selected replicates are split: Phage5 clusters with CON3, while Phage2 and Phage4 fall near, but distinct from, the Ancestor cluster. Ellipses represent 95% confidence intervals per population. CON3 separates from the other control replicates on PC1, consistent with ordinary replicate-to-replicate divergence over 35 days of independent serial passage.

The principal component analysis of all 19 samples revealed a clear separation of the experimental groups along the first two principal components, which together accounted for 59% of total transcriptomic variance (PC1: 40%; PC2: 19%), as shown in Figure 1. The iron-selected and phage/iron-selected populations were separated along the positive PC1 axis compared to the Ancestor and control populations, and their 95% confidence ellipses overlapped extensively, indicating that their gene expression patterns converged despite their different evolutionary histories. The phage-selected population occupied an intermediate-to-negative position on PC1: Phage5 clustered tightly with CON3, while Phage2 and Phage4 fell closer to, but did not overlap with, the Ancestor cluster, indicating that phage selection shifted the transcriptome away from the ancestral genomic background without approaching the iron-adapted populations. Within the control population, CON3 separated from CON1 and CON2 along PC1, falling instead near Phage5. As an independently propagated replicate lineage subjected to the same 35-day serial-passage regime as every other population in this experimental evolution study, CON3 is expected to show some degree of replicate-specific drift; however, its position on the PCA most likely reflects normal lineage-to-lineage variation rather than a technical artifact. PC2 (19% of variance) separated the Ancestor replicates (ANC1, ANC3, ANC4) from the FE/PF cluster along the negative axis, with the control and phage-selected samples occupying an intermediate position; ANC5 was the exception among the Ancestors, clustering instead with CON3 and Phage5. This pattern suggests that PC2 captures a combination of within-population replicate variation and a general divergence of the iron-adapted transcriptome from the founding population, rather than a phage-resistance-specific axis. Because CON3 fell apart from the other two control replicates on PC1, we performed a sensitivity analysis by re-running all pairwise comparisons with CON3 excluded. The DEG counts and the identity of significant genes were materially consistent between the full dataset and the CON3-excluded dataset. CON3 was therefore retained in the final analysis, as its inclusion did not change the overall pattern of results (see the Supplementary Materials). Its position on the PCA is best interpreted as ordinary within-lineage variation that arises over 35 days of independent serial passage, which is expected in any experimental evolution design with multiple replicate populations.

### 2.3. Hierarchical Clustering of the Top Differentially Expressed Genes Across All Experimental Groups

**Figure 2:**
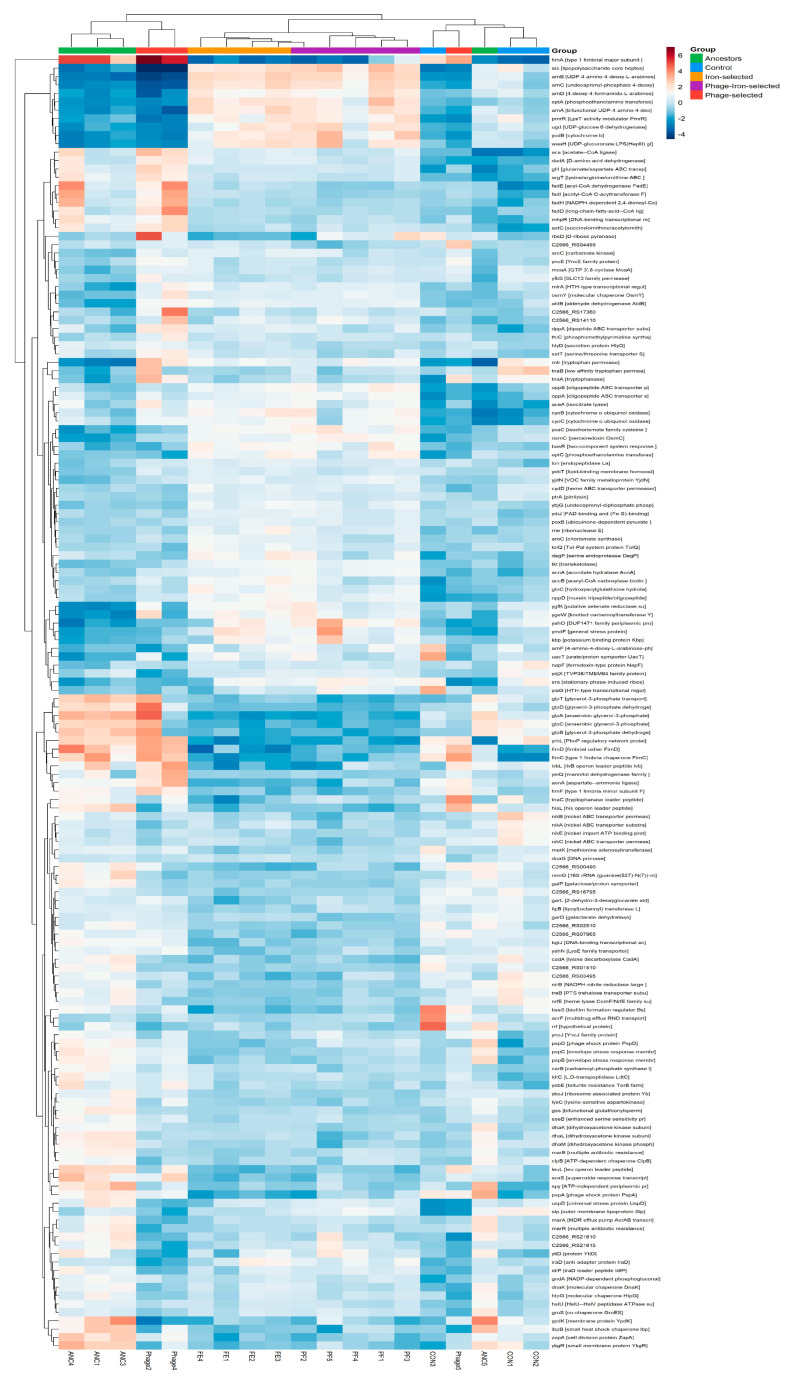
Heatmap of the top differentially expressed genes in *E. coli* B ATCC 11303 across all five experimental groups (Ancestor, control, phage-selected, FE, and PF). The color scale (red: upregulated; blue: downregulated) represents the log_2_ fold change (P_adj_ < 0.05, |log_2_FC| ≥ 1) relative to the row mean. Hierarchical clustering of the experimental groups and genes reveals a convergent lipid A/LPS-modification expression shared by the iron-selected and phage/iron-selected populations. In contrast, Ancestor and some phage-selected replicates showed an opposing expression pattern, while the remaining control and phage-selected samples occupied an intermediate position.

Hierarchical clustering of the top differentially expressed genes across the five experimental groups revealed three major expression patterns (Figure 2). One prominent pattern separated the iron-adapted populations, including the iron-selected (FE) and phage/iron-selected (PF) replicates, from most Ancestor, control, and phage-selected samples. The FE and PF populations showed strong relative enrichment of LPS-modification and envelope-remodeling genes, including *eptA*, *arnA*, *arnB*, *arnC*, *ugd*, *pmrR*, *waaH*, and related *arn*-regulon genes. This pattern is consistent with activation of lipid A/LPS remodeling pathways during adaptation to elevated iron(III) stress.

A second expression pattern was characterized by coordinated changes in metabolic genes, including *acs*, *fadE*, *fadI*, *fadH*, *fadD*, *gltI*, and *dadA*, suggesting that iron adaptation was also associated with shifts in fatty-acid and amino-acid metabolism. These metabolic changes were prominent in the iron-adapted populations but appeared secondary to the large envelope-remodeling signal.

A third pattern included samples that occupied an intermediate position between the strongly iron-adapted and ancestor-like pattern, including control, Ancestor, and phage-selected replicates. These samples showed less extreme expression of the LPS-modification cluster, consistent with their intermediate placement in the PCA. Across the heatmap, FE and PF samples clustered closely together, visually reinforcing their transcriptomic convergence. In contrast, phage-selected populations did not form a single uniform pattern; instead, some phage-selected replicates clustered closer to Ancestor populations, while others clustered closely to the control population. This suggests that phage selection alone produced a more heterogeneous transcriptional response than iron selection.

The color scale, which ranges from log_2_FC (P_adj_ < 0.05, |log_2_FC| ≥ 1) −5 to +6, shows that LPS-modification and envelope-remodeling genes represent one of the largest-magnitude signals in the dataset. The key genes in this category (*eptA*, *basR*, *arnA*, *arnB*, *arnC*, *ugd*, and *pmrR*) showed strong relative upregulation in the iron-selected and phage/iron-selected populations compared with the Ancestor, control, and phage-selected populations. This signal is particularly evident for genes such as *eptA*, *basR*, *arnA*, *arnB*, *arnC*, *ugd*, and *pmrR* and appears to exceed or match the magnitude of the fatty-acid and amino-acid metabolic shifts.

### 2.4. Pairwise Comparison of Differentially Expressed Genes of All Populations Within the Five Experimental Groups

**Figure 3:**
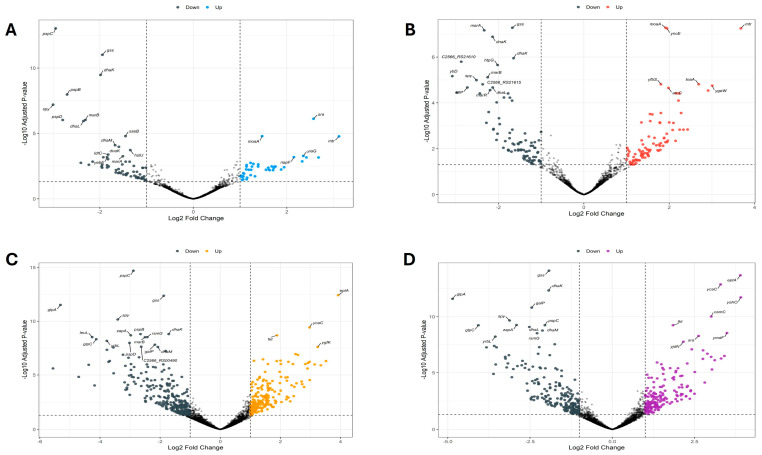
(**A**–**D**) Volcano plots of differentially expressed genes of the five experimental groups in pairwise comparison versus the Ancestor. (**A**) Control vs. Ancestor: 41 upregulated (blue) and 63 downregulated (dark grey) genes. (**B**) Phage-selected vs. Ancestor: 91 upregulated (red) and 86 downregulated (dark grey) genes. (**C**) Iron-selected (FE) vs. Ancestor: 201 upregulated (orange) and 281 downregulated (dark grey) genes. (**D**) Phage/iron-selected (PF) vs. Ancestor: 182 upregulated (purple) and 199 downregulated (dark grey) genes. Significance is defined by Padj < 0.05, |log_2_FC| ≥ 1.

When we compared the control population to the Ancestor population, we identified 104 differentially expressed genes, of which 41 were upregulated and 63 were downregulated (see Figure 3A). The *pspC*, *gss*, *dhaK*, *pspB*, *spy*, and *pspD*, which encode phage-shock-protein components, glutathione synthetase, and dihydroxyacetone-kinase subunits [18], are the most significantly downregulated genes, alongside *marB*, *dhaM*, *dnaK*, *ldtC*, *hslU*, *yebE*, and *marA*, whereas *sra*, *mtr*, *moaA*, *yiaG*, and *napF* genes were significantly upregulated. This shows that even growth in LB broth for 35 days without phage or iron selection produced a substantial transcriptional shift away from the Ancestor, with broad repression of stress-response and dihydroxyacetone-metabolism genes and induction of stationary-phase and molybdenum-cofactor biosynthesis genes. This indicates that the control population is not transcriptionally static relative to the Ancestor and must be taken into account when interpreting comparisons of the evolved populations.

Comparing the phage-selected population to the Ancestor, we identified 177 differentially expressed genes, 91 upregulated and 86 downregulated (Figure 3B). Downregulated genes included *marA*, *gss*, *dnaK*, *C2566_RS21610*, *htpG*, *dhaK*, *spy*, *marB*, *ytiD*, *idlP*, *marR*, and *dhaL*, many of which are stress-response, chaperone, and multiple-antibiotic-resistance (*mar*) regulon genes. Upregulated genes included *mtr*, *moaA*, *yncE*, *yfbS*, *tnaA*, *arcC*, and *ygeW*. Notably, none of the recognized iron-stress markers (*arnA/B/C*, *eptA*, *pmrR*, *basR*) appear among the phage-selected vs. Ancestor DEGs, indicating that, while phage selection does produce extensive transcriptional change relative to the founding population, this change occurs along a different axis than the iron-stress response and does not involve activation of the LPS-modification (*arn/eptA*) pathway that characterizes the iron-adapted populations.

A total of 482 differentially expressed genes were observed when comparing the iron-selected population (FE) to the Ancestor (ANC), of which 201 were upregulated and 281 were downregulated (Figure 3C). This extensive transcriptional rewiring reflects the intensity of the adaptive stress imposed by iron(III). The most strongly upregulated genes were *eptA*, *ycaC*, *tkt*, and *ygfK*, with *eptA* (lipid A phosphoethanolamine transferase) showing one of the most significant fold-changes and most significant adjusted *p*-values in the entire dataset. The most significantly downregulated genes were *glpA*, *pspC*, *gss*, *spy*, *leuL*, *glpC*, *zapA*, *pspB*, *rsmG*, *marB*, *yrbL*, *pspD*, *galP*, *dhaM*, and *dhaK*. The downregulation of *glpA*, *glpC*, and *galP* indicates broad repression of glycerol-3-phosphate utilization and galactose transport, while the downregulation of *pspB*, *pspC*, and *pspD* indicates that, unlike in the control population, the phage-shock protein response is actively suppressed rather than simply absent. The downregulation of *zapA* (a cell-division activator) and the upregulation of *eptA* together suggest that iron-adapted cells slow cell division while remodeling lipid A to reduce envelope permeability and limit further iron(III) influx.

When we compared the phage/iron(III)-selected population (PF) to its Ancestor, we found 381 DEGs, 182 upregulated and 199 downregulated (Figure 3D). The overlap with the FE vs. ANC comparison was extensive: the same top upregulated genes, *eptA*, *ycaC*, and *tkt*, recur here, joined by *yahO*, *osmC*, *sra*, *ymdF*, and *yjdN*, and the same top downregulated genes, *glpA*, *gss*, *dhaK*, *galP*, *spy*, *glpC*, *zapA*, *yrbL*, *pspC*, *dhaL*, *dhaM*, and *rsmG*, also recur. This transcriptomic profile, arising independently in populations with different selective histories (iron selection alone vs. phage selection, followed by iron selection), is the first indication of the convergent iron-stress transcriptional rewiring that is examined directly in the PF vs. FE comparison.

**Figure 4:**
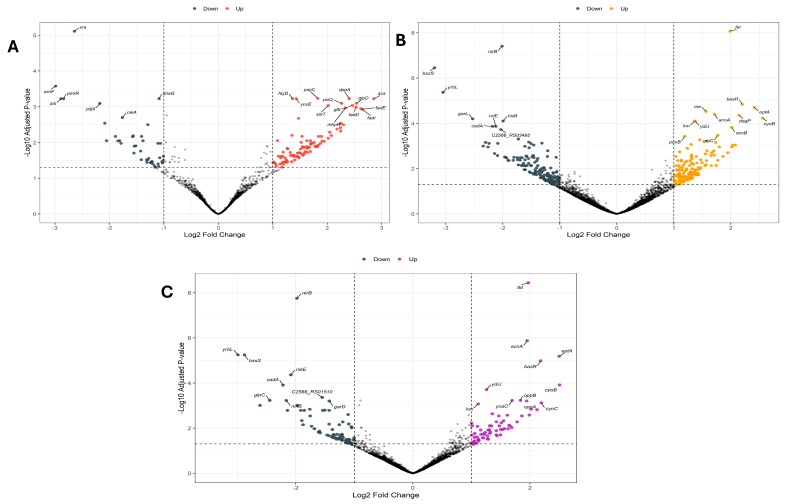
(**A**–**C**) Volcano plots of (**A**) phage-selected vs. control (139 DEGs: 96 upregulated, 43 downregulated), (**B**) iron-selected (FE) vs. control (286 DEGs: 131 upregulated, 155 downregulated), and (**C**) phage/iron-selected (PF) vs. control (145 DEGs: 70 upregulated, 75 downregulated). The shared upregulation of *eptA*, *basR*, *acnA*, and *tkt*, and shared downregulation of *nirB*, *yrbL*, and *bssS* in both the FE vs. CON and PF vs. CON panels illustrate the convergent iron-stress transcriptional signature in the two iron-adapted populations.

Pairwise comparisons between the evolved and the control populations also revealed substantial transcriptional divergence. In the phage-selected vs. control comparison, 139 DEGs were identified (96 upregulated, 43 downregulated; Figure 4A). The *sra*, *arnF*, *pmrR*, *ais*, *ydjX*, *nikA*, and *dnaG* genes were significantly downregulated. The presence of *arnF* and *pmrR* among the downregulated genes is notable: these LPS-modification genes are lower in the phage-selected population than in the control, indicating that the phage-selected population does not activate the iron-stress/*arn* regulon, while the control, which derives from the same 35-day culture period as the other evolved lines, shows comparatively higher baseline expression of these genes. The *hlyD*, *yncE*, *pspC*, *yeiQ*, *sstT*, *dppA*, *gltI*, *fadD*, *mhpR*, *glpD*, *fadE*, *fadI*, and *acs* genes were upregulated, indicating an increased fatty-acid and dipeptide/oligopeptide transport and catabolism capacity in the phage-selected population relative to the control.

The iron-selected vs. control comparison yielded 286 DEGs (131 upregulated, 155 downregulated; Figure 4B), confirming that the iron-stress transcriptional signature observed relative to the Ancestor is not simply an artifact of comparing to the Ancestor baseline. Downregulated genes included *bssS*, *nirB*, *yrbL*, *garL*, *nrfE*, *cadA*, *treB*, and *C2566_RS03495*. Upregulated genes included *tkt*, *lon*, *rne*, *ydiJ*, *acnA*, *basR*, *degP*, *eptA*, *cyoB*, *poxB*, *accB*, and *gloC*. The recurrence of *eptA* and *basR* among the top upregulated genes in both the FE vs. ANC and FE vs. CON comparisons confirms that activation of the BasSR-regulated lipid A modification pathway is a robust feature of the iron-selected transcriptome, independent of which baseline population it is compared against.

The phage/iron-selected vs. control comparison showed 145 DEGs (70 upregulated, 75 downregulated; Figure 4C), again closely mirroring the FE vs. CON comparison. Downregulated genes included *nirB*, *yrbL*, *bssS*, *cadA*, *nikE*, *glpC*, *C2566_RS01510*, *nikB*, and *garD*. Upregulated genes included *tkt*, *acnA*, *basR*, *eptA*, *ydiJ*, *lon*, *ycaC*, *oppB*, *oppA*, *cyoB*, and *cyoC*. The near-complete overlap in identity and direction of the top DEGs between the FE vs. CON and PF vs. CON comparisons (*tkt*, *acnA*, *basR*, *eptA*, and *ydiJ* upregulated; *nirB*, *yrbL*, *bssS*, and *cadA* downregulated in both) provides additional, independent evidence that the iron-selected and phage/iron-selected populations have converged on the same transcriptional program, regardless of whether the Ancestor or the control is used as the reference.

**Figure 5:**
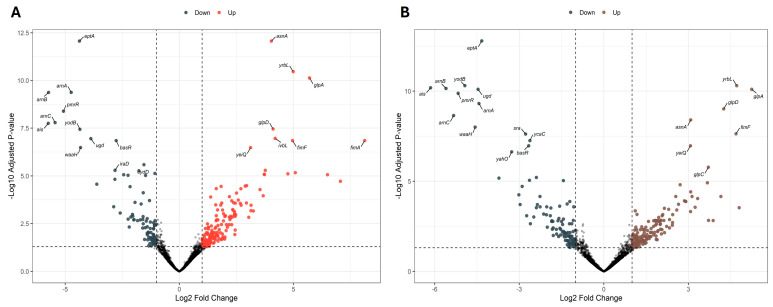
(**A**,**B**) Volcano plots of (**A**) phage-selected vs. iron-selected (FE) populations (255 DEGs: 168 upregulated, 87 downregulated) and (**B**) phage-selected vs. phage/iron-selected (PF) populations (262 DEGs: 155 upregulated, 107 downregulated). *eptA* and the *arn* operon genes are the most significantly downregulated genes in phage-selected relative to both iron-adapted populations, while fimbrial (*fimA*, *fimF*) and glycerol-3-phosphate (*glpA*, *glpC*, *glpD*) genes are the most strongly upregulated.

Comparing the phage-selected population directly with each iron-adapted population revealed the largest pairwise differences in the entire dataset, consistent with phage and iron resistance representing distinct strategies. In the phage-selected vs. iron-selected comparison, 255 DEGs were identified (168 upregulated, 87 downregulated; Figure 5A). The most strongly downregulated genes in the phage-selected population (i.e., higher in FE) were *eptA*, *arnA*, *arnB*, *pmrR*, *arnC*, *ais*, *yodB*, *ugd*, *waaH*, and *basR*—essentially the complete set of *arn* operon and lipid A-modification genes identified above as iron-stress markers. The most strongly upregulated genes in the phage-selected population (i.e., higher in Phage than FE) were *asnA*, *yrbL*, *glpA*, *glpD*, *yeiQ*, *ivbL*, *fimF*, and *fimA*, with the type 1 fimbrial genes *fimA* and *fimF* showing among the largest fold-changes observed in any comparison in this study. This indicates that the phage-selected population retains or re-activates fimbrial adhesin expression that is strongly repressed in the iron-adapted background.

The phage-selected vs. phage/iron-selected comparison produced a near-identical pattern, with 262 DEGs (155 upregulated, 107 downregulated; Figure 5B). Downregulated genes in the phage-selected population included *eptA*, *arnB*, *yodB*, *ugd*, *pmrR*, *arnC*, *arnA*, *waaH*, *sra*, *ycaC*, *yahO*, and *basR*, again matching the iron-stress/*arn* signature. Upregulated genes included *yrbL*, *glpA*, *glpD*, *asnA*, *yeiQ*, *glpC*, and *fimF*. The fact that the phage-selected population differs from both FE and PF by essentially the same set of genes, while FE and PF do not differ from each other at all, reinforces the conclusion that there is a single, shared iron-adapted transcriptional state (occupied jointly by FE and PF) that is clearly distinguished from the phage-selected state, rather than three separate states.

**Figure 6:**
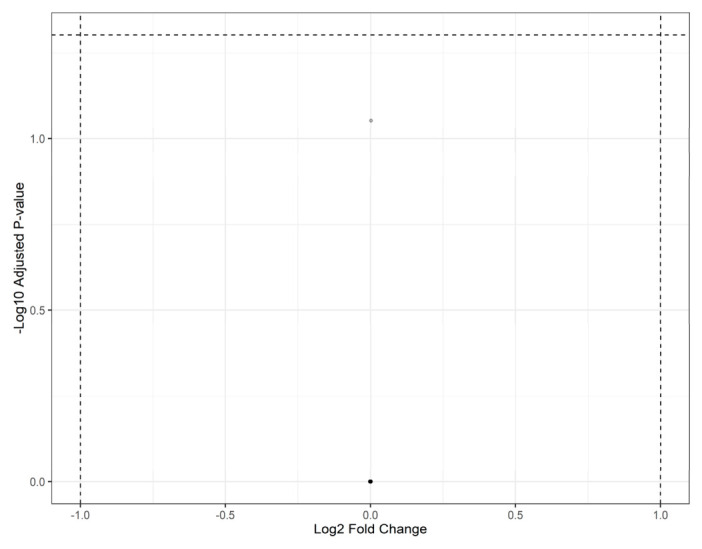
Volcano plot of the phage/iron-selected (PF) vs. iron-selected (FE) comparison. Zero genes are differentially expressed at significance (P_adj_ < 0.05, |log_2_FC| ≥ 1), despite each population individually showing hundreds of DEGs relative to the Ancestor and control.

Despite the hundreds of genes that distinguish each iron-adapted population from the Ancestor and from the control, the direct comparison between the phage/iron-selected and iron-selected populations yielded zero differentially expressed genes (P_adj_ < 0.05, |log_2_FC| ≥ 1; Figure 6). This is a striking result: two populations that each show 286–482 DEGs relative to a common baseline are statistically indistinguishable from one another at the same significance threshold. This provides strong evidence that, regardless of whether iron resistance was acquired directly (FE) or following prior phage selection (PF), the resulting transcriptional state is the same. Because this comparison is unaffected by the change in DEG counts elsewhere in the dataset and reproduces the result obtained in our original analysis, it is the most robust finding of this study and forms the basis for the convergence/canalization argument developed in the Section 3.

## 3. Discussion

Gene expression studies were performed on *Escherichia coli* B populations derived from the 35-day experimental evolution delineated in Ezeanowai et al. [15], which include five experimental groups denoted as Ancestor (ANC), control (CON), phage-selected (Phage), iron(III)-selected (FE), and phage/iron(III)-selected (PF). The sequencing reads from the five experimental groups were aligned to the *E. coli* B ATCC 11303 reference genome, and pairwise comparisons, principal component analysis, and hierarchical clustering between groups were performed to characterize differential gene expression profiles. Principal component analysis (Figure 1) showed that the iron-selected and phage/iron-selected populations clustered closely, with overlapping 95% confidence ellipses, suggesting their gene expression patterns were similar despite different evolutionary histories. This aligns with LaCroix et al. [19], who reported that bacteria exposed to the same stress can show similar gene expression patterns despite different evolutionary trajectories.

The hierarchical clustering (Figure 2) revealed three major expression patterns. One pattern separated the iron-adapted populations, FE and PF, from most Ancestor, control, and phage-selected populations, with the FE/PF replicates showing strong relative upregulation of *eptA*, *arnA*, *arnB*, *arnC*, *ugd*, *pmrR*, and *waaH*, the lipid A/LPS-modification genes of the *arn* regulon, consistent with active lipid A remodeling during iron(III) adaptation. A second pattern, also prominent in FE and PF, reflected coordinated upregulation of fatty-acid and amino-acid metabolism genes (*acs*, *fadE*, *fadI*, *fadH*, *fadD*, *gltI*, *dadA*), though this signal was secondary in magnitude to the envelope-remodeling pattern. A third pattern comprised populations occupying an intermediate position between the strongly iron-adapted and Ancestor-like profiles, including several control, Ancestor, and phage-selected replicates, with less extreme expression of the LPS-modification cluster. Notably, the phage-selected replicates did not cluster uniformly: some cluster closer to the Ancestor-like profile, while others fell with the intermediate populations, indicating that, unlike the iron-adapted populations, phage selection alone did not produce a single, homogeneous transcriptional response across replicates, even though, as shown in Figure 3B and Table 1, the phage-selected population as a whole differs from the Ancestor at 177 genes outside this LPS-modification gene set.

The pairwise comparisons showed significant upregulation of the *arn* operon (*arnA*, *arnB*, *arnC*, *arnD*), together with *eptA*, *pmrR*, and *basR*, in iron-selected and phage/iron-selected populations compared with the Ancestor, control, and phage-selected populations. The arn pathway, together with the BasSR-regulated lipid A phosphoethanolamine transferase *eptA*, adds positively charged 4-amino-4-deoxy-L-arabinose and phosphoethanolamine groups to lipid A, which reduces the net negative charge of the outer membrane and increases resistance to cationic stress, including iron(III) and cationic antimicrobial peptides [20,21,22,23,24]. This pathway is typically activated by the PmrAB and BasSR two-component systems in response to iron and low pH [22,25,26], and its strong, consistent upregulation across the FE vs. ANC, FE vs. CON, PF vs. ANC, and PF vs. CON comparisons indicates that lipid A remodeling is the dominant transcriptional response to iron(III) stress in this system. This supports the phenotypic results from Ezeanowai et al. [15], who showed that only iron-adapted populations tolerated 1750 mg/L Fe(III). At the genomic level, regulatory genes in this same network (*qseB*, *qseC*, *basR*) were found to be under selective sweeps in the iron-selected and phage/iron-selected backgrounds [15], and their corresponding increase in downstream *arn/eptA* expression shows that expression-level regulation is a key component of the adaptive response, consistent with the mutations translating into a coherent downstream transcriptional program rather than acting only at the level of protein sequence [16,17].

Alongside this membrane-remodeling signature, the iron-adapted populations also downregulated genes involved in glycerol-3-phosphate metabolism (*glpA*, *glpC*, *glpD*), the products of the aerobic and anaerobic glycerol-3-phosphate dehydrogenase operons [27], consistent with reports that BasSR-regulated iron responses are coupled to repression of glycerol-3-phosphate and other TCA-linked metabolic genes [25,28], and the phage-shock-protein stress response (*pspB*, *pspC*, *pspD*), while upregulating fatty-acid and amino-acid catabolism genes (*acs*, *fadD*, *fadE*, *fadI*, *gltI*, *dadA*). One interpretation is that, because the phage-shock-protein response (Psp) is itself triggered by inner-membrane stress and dissipation of the proton motive force [29], its suppression in iron-adapted cells alongside strong activation of the lipid A-remodeling pathway suggests that proactive envelope remodeling may reduce the inner-membrane stress that would otherwise trigger the Psp response, while the concurrent shift toward fatty-acid and amino-acid catabolism could supply the additional carbon and energy needed to sustain this membrane-modification program. *tkt* (transketolase), one of the most significantly upregulated genes in both the FE vs. CON and PF vs. CON comparisons, links the pentose phosphate pathway to this same metabolic shift; the non-oxidative branch of this pathway, in which transketolase participates, supports the reserve flux capacity that allows the oxidative branch to rapidly increase NADPH supply during oxidative stress [30], which may help manage iron-induced oxidative damage. However, it should be noted that RNA-seq identifies transcriptional associations rather than functional necessity; whether upregulation of the *arn/eptA* pathway is required for iron resistance or represents a correlated but non-essential response cannot be determined from transcriptomic data alone. Targeted functional validation, such as gene knockouts or overexpression experiments under iron stress conditions, is necessary to establish causal roles for these genes in the observed resistance phenotype.

Another important finding in this study is that iron-selected and phage/iron-selected populations have identical transcriptomic profiles (0 DEGs in PF vs. FE), as shown in Figure 6, even though their genomic profiles are clearly different (iron(III)-selected populations had sweeps in *qseB*, *qseC*, and *ydbD*, while phage/iron populations had sweeps in *basR*, *aroK*, and *rseB*) [15]. This provides strong evidence that their gene expression has fully converged. In Ezeanowai et al. [15], iron(III)-selected populations had sweeps in *qseB*, *qseC*, and *ydbD*, while phage/iron populations had sweeps in *basR*, *aroK*, and *rseB*, with no overlap. Despite these different regulatory mutations, both populations end up with the same downstream transcriptional changes. This situation, in which distinct genomic changes yield the same transcriptomic outcome, is called systems-level canalization, a concept originally developed to explain how gene regulatory networks can buffer phenotypic outcomes against both genetic and environmental perturbation [31], and has been observed in other experimental evolution studies [16,19]. Unequal replicate numbers across experimental groups (four for FE, five for PF, but three for the phage-selected and control populations) could in principle limit statistical power and produce spuriously low DEG counts in some comparisons, since biological replicate number is generally a stronger determinant of RNA-seq power than sequencing depth [32]. However, this concern does not appear to explain the PF vs. FE result: the same unequal-replicate design detected 482, 381, 286, and 145 DEGs in the FE vs. ANC, PF vs. ANC, FE vs. CON, and PF vs. CON comparisons, respectively, demonstrating that the pipeline has ample power to detect differences of this kind when they exist. The complete absence of DEGs specifically in the PF vs. FE comparison, despite this demonstrated sensitivity, strengthens rather than weakens the case that the convergence between these two populations reflects genuine biological canalization rather than an artifact of limited power. In *E. coli*, two-component regulatory systems, such as *PmrAB*, *QseBC*, and *BasSR*, share overlapping regulons and can activate the same target genes [21,25,33,34]. So, mutations in *qseB* (QseBC system) in iron-selected populations and *basR* (BasSR system) in phage/iron-selected populations may both lead to similar levels of *arn* operon and *eptA* expression through their own signaling pathways [21,33,34]. This convergence is important because it means that, even though iron-selected and phage/iron-selected populations appear the same at the transcriptomic and phenotypic levels, their evolutionary histories (such as phage pre-selection) cannot be identified by transcriptomic profiling alone. However, differences in their basal mutations and potential fitness costs could lead to distinct responses to future stress [1,17].

Phage-selected populations showed substantial transcriptional change relative to both the Ancestor (177 DEGs) and the control (139 DEGs), but this change occurred along a different axis than the iron-stress response and aligns with the genomic data in an important respect: none of the canonical iron-stress markers (the *arn* operon, *eptA*, *basR*) were activated in the phage-selected population. The genomic analysis showed that T4 phage resistance was conferred by loss-of-function mutations or deletions in *waaA*, *waaG*, and *rfaQ*, which encode enzymes involved in LPS core biosynthesis [15,35]. This prevents the T4 tail fibers from attaching, which can confer phage resistance through structural receptor loss rather than through adoption of the iron-stress regulon [15,35,36,37]. Instead, the genes that distinguish the phage-selected population from the Ancestor and control are dominated by the multiple-antibiotic-resistance regulon (*marA*, *marB*, *marR*) and chaperone genes (*dnaK*, *htpG*), all downregulated relative to the Ancestor [38,39], alongside upregulation of *mtr* (tryptophan permease) and *moaA* (molybdenum-cofactor biosynthesis). The downregulation of the *mar* regulon, which controls a multidrug-efflux pump and is normally induced by a range of antibiotics and oxidative stressors [38,39], is notable given that this population also lost LPS core structure; reduced baseline *mar* expression in a strain with an already-altered envelope could have implications for its susceptibility to other antimicrobials, a question directly relevant to the combination-therapy rationale motivating this study. This pattern shows that phage resistance and iron resistance are transcriptionally distinct adaptations: both involve hundreds of gene expression changes relative to the founding population, but they occur on largely non-overlapping gene sets, with the lipid A/LPS-modification axis specific to iron stress and the multidrug-resistance/chaperone axis specific to, or at least more pronounced in, the phage-selected background.

Our DNA-seq analyses showed that exposure to elevated iron(III) results in iron-specific mutations, such as mutations in *qseB*, *qseC*, and *fieF*, leading to increased expression of genes for membrane modification (*arnA*, *arnB*, *arnC*, *arnD*, *eptA*, *pmrR*) and altering metabolism, with downregulation of glycerol-3-phosphate utilization (*glpA*, *glpC*, *glpD*) and upregulation of fatty-acid/amino-acid catabolism (*acs*, *fadD*, *fadE*). In phage/iron-selection populations (populations that first faced phage selection and then iron selection), the same gene expression change occurs but through mutations in *basR* and *aroK*, which are different ways into the same regulatory network. In contrast, phage resistance functions by altering the receptor (via a *waaA*/*waaG* deletion) and is associated with a largely separate transcriptional response (the *mar* regulon and chaperone genes, as described above) rather than the *arn/eptA* pathway, suggesting that the convergent transcriptomes of the iron-selected and phage/iron-selected populations are specific to the iron-stress regulatory network and would not necessarily extend to a new, third stressor that interacts differently with their distinct genomic backgrounds.

Our DNA and RNA sequencing analyses both point to the outer membrane as a recurring target of selection. In the RNA-seq analyses, the *arnA*, *arnB*, *arnC*, and *arnD* genes, which together synthesize and attach 4-amino-4-deoxy-L-arabinose to lipid A, and *eptA*, which adds phosphoethanolamine to lipid A [7,24], were the most strongly upregulated genes in both the FE vs. ANC and PF vs. ANC comparisons. The same genes were significantly downregulated when the phage-selected population was compared directly to FE or PF (Figure 5), confirming that this lipid A-modification rewiring is specific to the iron-stress response and is not shared by the phage-selected population.

In our DNA-seq analyses, we observed large deletions in the *waaA* and *waaG* genes in phage-resistant populations, which shorten the LPS core [15,35]. The repeated focus on membrane-related genes under different selective pressures shows that the outer membrane is both a main target and a flexible defense in bacterial adaptation to environmental pressures. The increased expression of the type 1 fimbrial genes *fimA* and *fimF* in the phage-selected population, relative to both FE and PF, is striking because type 1 fimbriae are surface adhesins whose expression is phase-variable and responsive to envelope and other environmental stress signals [40]. Their selective retention or re-activation in the phage-selected background, which lost LPS core structure through *waaA/waaG* deletion, but not in the iron-adapted populations, which actively remodel lipid A through the *arn/eptA* pathway, suggests that the two resistance strategies place opposite demands on surface-structure expression: iron adaptation favors a heavily lipid-A-modified, lower-adhesin surface, whereas phage resistance, having already lost the phage receptor, is free to retain or increase fimbrial expression. This finding illustrates how the specific route to resistance, structural receptor loss versus active regulatory remodeling, leaves a distinct and identifiable signature on surface-associated gene expression even when the resistant populations are otherwise compared under the same growth conditions.

## 4. Materials and Methods

### 4.1. Experimental Populations and RNA Extraction

The *Escherichia coli* B populations utilized for RNA-seq were derived from the 35-day experimental evolution delineated in Ezeanowai et al. [15]. The genome of *E. coli* B (ATCC 11303) is composed of 4,622,284 base pairs, encoding 4494 genes. The Ancestor populations were derived by culturing pure colonies of *E. coli* B (ATCC 11303) (see Ezeanowai et al. 2025 [15]). The control populations were set up by transferring 0.1 mL of overnight Ancestor culture in sterile 50 mL flasks containing 9.9 mL of LB broth. The phage-selected populations were evolved by inoculating 0.1 mL of T4-phage and 0.1 mL of each *E. coli* B population from the Ancestor culture in 9.8 mL of LB broth (Fisher Scientific, Fair Lawn, NJ, USA). The iron(III)-selected populations were evolved by inoculating 0.1 mL of the overnight culture (Ancestor culture) into 9.9 mL of LB broth containing 1500 mg/L iron(III) sulfate (Thermo Fisher Scientific, Waltham, MA USA). The phage-iron(III)-selected populations were evolved by inoculating 0.1 mL of overnight culture of the phage-selected populations (phage-selected culture) into 9.9 mL of LB broth containing 1500 mg/L iron(III) sulfate (see Ezeanowai et al. 2025 [15], section 4.1). All the control (CON), phage-selected (Phage), iron(III)-selected (FE), and phage/iron(III)-selected (PF) populations were serially transferred for 35 days except for the Ancestor population, which was only grown for 24 h (25 mL) and was stored in 50% (*v*/*v*) glycerol (Thermo Fisher Scientific, Dreieich, Germany) at a 1:1 ratio and frozen at −80 °C until needed for analysis. The reference genome (ATCC 11303) can be accessed at https://www.ncbi.nlm.nih.gov/datasets/genome/GCA_034424725.1/ (accessed on 5 January 2026).

RNA was extracted from the Ancestor (ANC), control (CON), phage-selected (Phage), iron(III)-selected (FE), and phage/iron(III)-selected (PF) populations using TRIzol (Thermo Fisher Scientific Inc.), to obtain pure, high-quality RNA.

### 4.2. Library Preparation and Sequencing

The total RNA extraction of the Ancestors, control, phage-selected, iron(III)-selected, and phage/iron(III) populations after 35 days of culture, as delineated in Ezeanowai et al. [15], was carried out using TRIzol™ Reagent to obtain pure, high-quality RNA (Thermo Fisher Scientific Inc.), following the manufacturer’s instructions. The RNA concentrations were normalized according to the Illumina total RNA library prep protocol using the QuantiFluor^®^ dsDNA system (Illumina, San Diego, CA, USA). The total RNA libraries were prepared using the Illumina total RNA Prep kit, and strict adherence was maintained to the Illumina total RNA library prep protocol. The samples were sequenced using the Illumina NextSeq 2000 sequencing platform. The depth of coverage of the sequencing runs ranged from 40 to 100×, with some exceeding, and the read length per sample was 2 × 50 bp. Reads that indicate a difference between the sample and the reference genome that cannot be resolved to describe precise transcriptomic changes were removed. Four replicates of iron(III)-resistant populations were successfully sequenced, designated FE1, FE2, FE3, and FE4. Three replicates of phage-resistant populations were successfully sequenced, designated phage2, phage4, and phage5. Five replicates of phage/iron(III) resistant populations were codenamed PF1, PF2, PF3, PF4, and PF5, and three controls were sequenced and designated CON1, CON2, and CON3. Four replicates of Ancestor populations were successfully sequenced, designated ANC1, ANC3, ANC4, and ANC5.

### 4.3. Bioinformatics Analysis

Trimmomatic v0.39 (Usadel Lab, Aachen, Germany) [41] was used to quality-trim the raw reads, and HISAT2 v2 (Johns Hopkins University, Baltimore, MD, USA) [42] was used to align Fastq reads to the *E. coli* B ATCC 11303 reference genome (GCA_034424725.1; genome size: 4,622,284 bp). All evolved populations and the Ancestors were aligned to the reference genome (*Escherichia coli* B ATCC 11303) and then compared to the ancestral population. We used featureCounts (Subread v2.0.6; Walter and Eliza Hall Institute of Medical Research, Melbourne, VIC, Australia) [42] to count the reads. We used DESeq2 v1.38.0 (European Molecular Biology Laboratory, Heidelberg, Germany) [43] in R (R Foundation for Statistical Computing, Vienna, Austria) [44] for differential expression analysis. The Wald test and the Benjamini–Hochberg multiple testing correction were used [45]. The significance thresholds were set at |log_2_FC| ≥ 1 and p_adj_ < 0.05. We performed principal component analysis on stabilized count data using the DESeq2 variance-stabilizing transformation (vst). EnhancedVolcano v 1.26.0 (Bioconductor Project, Boston, MA, USA) [46] was used to generate volcano plots. Heatmaps were generated with pheatmap v1.0.12 (University of Tartu, Tartu, Estonia) [47], applying hierarchical clustering (Ward’s method, Euclidean distance) [48].

## 5. Conclusions

Iron stress induces extensive transcriptional rewiring. There are 381–482 DEGs between the iron-selected and phage/iron-selected populations compared to the Ancestor and 145–286 DEGs compared to the control. These differences are dominated by lipid A/LPS membrane modification (*arnA*, *arnB*, *arnC*, *arnD*, *eptA*, *pmrR*, *basR*), reduced glycerol-3-phosphate metabolism (*glpA*, *glpC*, *glpD*), and a shift toward fatty-acid and amino-acid catabolism (*acs*, *fadD*, *fadE*, *fadI*, *gltI*). The *arn* operon and *eptA* are robust transcriptional markers of iron selection: they are significantly upregulated in the iron-adapted populations across every comparison against the Ancestor and the control, and they are the significantly downregulated genes when the phage-selected population is compared directly to either iron-adapted population. This suggests that they could be strong, reproducible transcriptomic markers of iron stress in *Escherichia coli* strain B.

Even though the iron-selected and phage/iron-selected populations have different mutational profiles (*qseB/qseC* in iron-selected vs. *basR/aroK* in phage/iron-selected), they exhibit the same transcriptomic phenotype (0 DEGs in phage/iron-selected vs. iron-selected), indicating that regulatory networks canalize onto a shared expression pattern, a form of functional epistasis. This convergence is robust: it persists even though each population individually shows hundreds of DEGs relative to the Ancestor and control, demonstrating that the absence of differences between FE and PF is not an artifact of limited statistical power. Phage resistance, by contrast, was readily detectable in the transcriptome, with the phage-selected population differing from the Ancestor at 177 genes and from the control at 139 genes but along an axis distinct from the iron-stress response and centered instead on the multiple-antibiotic-resistance (*mar*) regulon and chaperone genes. This is consistent with phage resistance arising primarily from alteration of a structural receptor (LPS core truncation via *waaA/waaG* loss) needed for phage binding while still triggering a broad, phage-specific transcriptional response distinct from iron adaptation.

Compared to both the Ancestor and the iron-adapted populations, the phage-selected population showed increased *fimA* and *fimF* (type 1 fimbrial genes) and decreased *arn/eptA* pathway expression. This indicates that the route taken to resistance, structural receptor loss for phage resistance versus active lipid A remodeling for iron resistance, leaves distinguishable and largely non-overlapping transcriptional signatures, aligning with the genomic epistasis detailed by Ezeanowai et al. [15].

These findings show that the exposure sequence of environmental stressors affects both genomic and transcriptomic adaptation and that a converged, near-identical transcriptome between two populations (FE and PF) can mask distinct underlying mutational paths to that same state. They also show that resistance to a structural threat (phage) and resistance to a chemical/oxidative threat (iron(III)) draw on substantially different parts of the transcriptome, with limited evidence of a shared, generic “stress response.”

This study is important because it shows how *E. coli* B responds to high iron(III) levels through a coordinated transcriptional response: the *arn* operon and *eptA* remodel lipid A to reduce envelope permeability and cationic susceptibility, while glycerol-3-phosphate metabolism is downregulated and fatty-acid/amino-acid catabolism is upregulated, plausibly to supply the resources needed to sustain this membrane-modification program. It also revealed a compelling example of system-level canalization, shown in the full transcriptomic convergence between the iron-selected and phage/iron-selected populations (0 DEGs in PF vs. FE), despite the fact that the underlying mutations and the route by which each population reached the iron-resistant phenotype differ. Because RNA-seq alone cannot establish whether the genes identified here are functionally required for resistance, future work combining targeted knockouts or overexpression with phenotypic iron-tolerance assays will be needed to move from this descriptive, transcriptome-wide picture to a mechanistic one.

### Future Directions

Future studies should functionally evaluate whether *arnA*, *arnB*, *arnC*, *arnD*, or *eptA* directly contribute to the iron-resistance phenotype, for example, by deleting these genes individually or in combination in the ancestral background and testing iron(III) tolerance or by transducing *qseB* or *basR* mutations into the ancestral background and asking whether they are sufficient to reproduce the downstream *arn/eptA* expression signature. Simultaneously, detailed examination of membrane remodeling, with a focus on chemical modifications of the LPS layer as determined by mass spectrometry, will help inform whether adaptive resistance is driven by the structural modifications predicted by the transcriptomic data. Given that the phage-selected population showed reduced expression of the *mar* multidrug-resistance regulon, future work should also directly test the antibiotic susceptibility of phage-resistant strains, since reduced baseline efflux-pump expression in an LPS-truncated background could affect their suitability for phage–antibiotic combination therapy.

## Figures and Tables

**Table 1:** Summary of differentially expressed genes across all pairwise comparisons (P_adj_ < 0.05, |log_2_FC| ≥ 1).

Comparison	Up	Down	Notable Downregulated	Notable Upregulated
CON vs. ANC	41	63	*pspC*, *gss*, *dhaK*, *pspB*, *spy*	*sra*, *mtr*, *moaA*, *yiaG*, *napF*
FE vs. ANC	201	281	*glpA*, *pspC*, *gss*, *spy*, *leuL*	*eptA*, *ycaC*, *tkt*, *ygfK*
PF vs. ANC	182	199	*glpA*, *gss*, *dhaK*, *galP*, *spy*	*eptA*, *ycaC*, *yahO*, *tkt*, *osmC*
Phage vs. ANC	91	86	*marA*, *gss*, *dnaK*, *htpG*, *dhaK*	*mtr*, *moaA*, *yncE*, *tnaA*, *ygeW*
FE vs. CON	131	155	*bssS*, *nirB*, *yrbL*, *garL*, *nrfE*	*tkt*, *basR*, *eptA*, *acnA*, *cyoB*
PF vs. CON	70	75	*nirB*, *yrbL*, *bssS*, *cadA*, *nikE*	*tkt*, *acnA*, *basR*, *eptA*, *ydiJ*
PF vs. FE	0	0	*—*	*—*
Phage vs. CON	96	43	*sra*, *arnF*, *pmrR*, *ais*, *ydjX*	*hlyD*, *yncE*, *pspC*, *yeiQ*, *acs*
Phage vs. FE	168	87	*eptA*, *arnA*, *arnB*, *pmrR*, *arnC*	*asnA*, *yrbL*, *glpA*, *glpD*, *fimA*
Phage vs. PF	155	107	*eptA*, *arnB*, *yodB*, *ugd*, *pmrR*	*yrbL*, *glpA*, *glpD*, *asnA*, *fimF*

## Data Availability

The SRA accession number for RNA sequencing data from this study is PRJNA1471254 for the Ancestor, iron(III)-selected, phage-selected, phage/iron(III)-resistant, and control populations.

## Supplementary Materials

The following supporting information can be downloaded at: https://www.mdpi.com/article/10.3390/antibiotics15070684/s1. The supplementary material includes original RNAseq analysis data, providing detailed information on gene descriptions, and expression values within the context of the study. Volcano plot, heatmap, and PCA plots differential gene expressions of the experimental populations. Sensitivity analysis of the CON3. This data supports the findings discussed in the main text by offering insights into the transcriptomic profile supporting resistance and adaptation in the populations observed in the study.

## Abbreviations

ANC	Ancestor
CON	control
PF	phage/iron(III)
FE	iron(III)
DEGs	differentially expressed genes
