# Supplementary figures and images for: Transcriptome Profiling of Escherichia coli B During Sequential Adaptation to T4 Phage and Iron(III) Stress
Source: Antibiotics (Basel). 2026 Jul 13;15(7):684. doi: 10.3390/antibiotics15070684 (PMC13405929; doi:10.3390/antibiotics15070684)

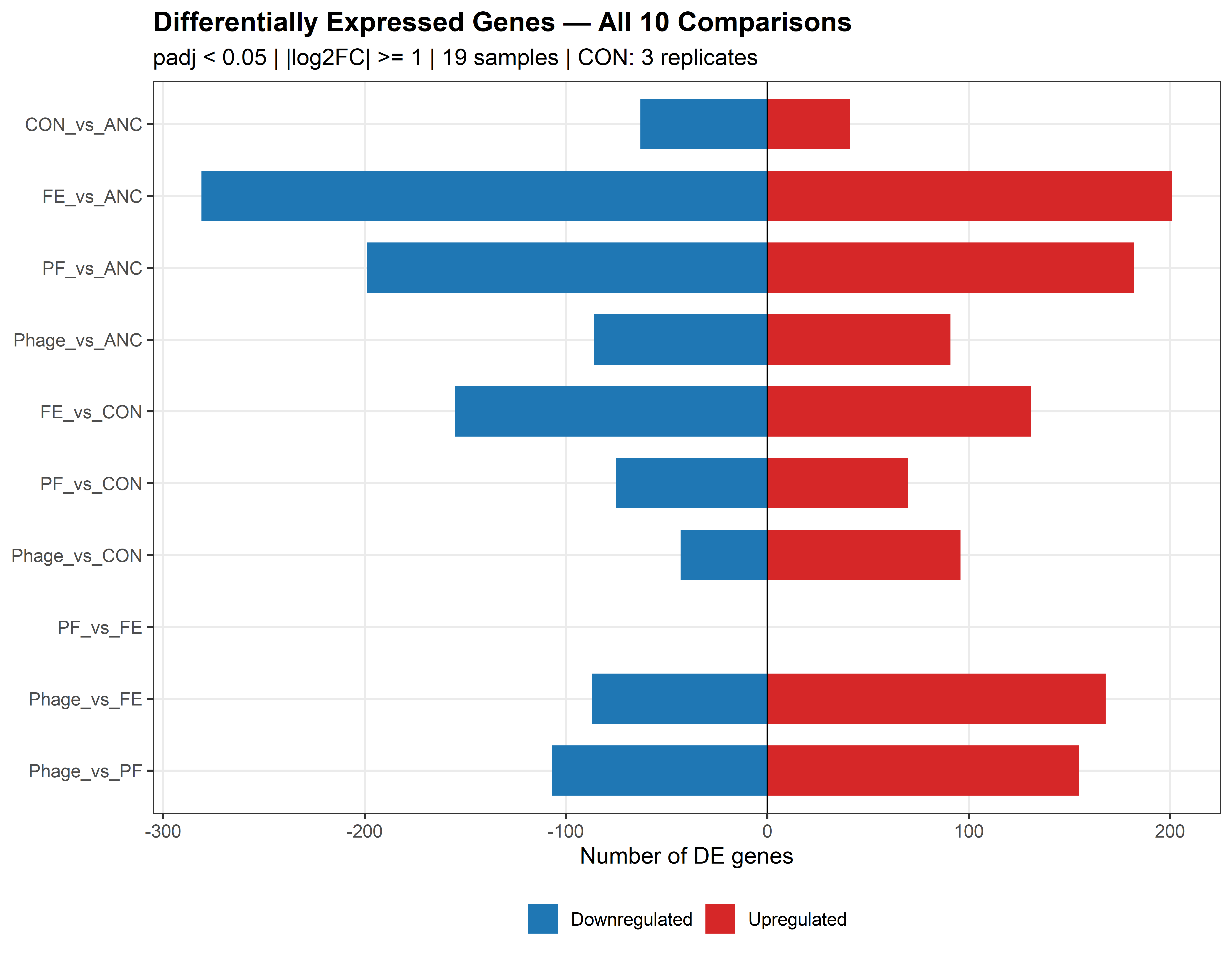

Supplement: Supplementary file 1 [file antibiotics-15-00684-s001.zip › SUPPLEMENTARY FOLDER/analysis_CON3/DE_gene_counts_barplot.png]

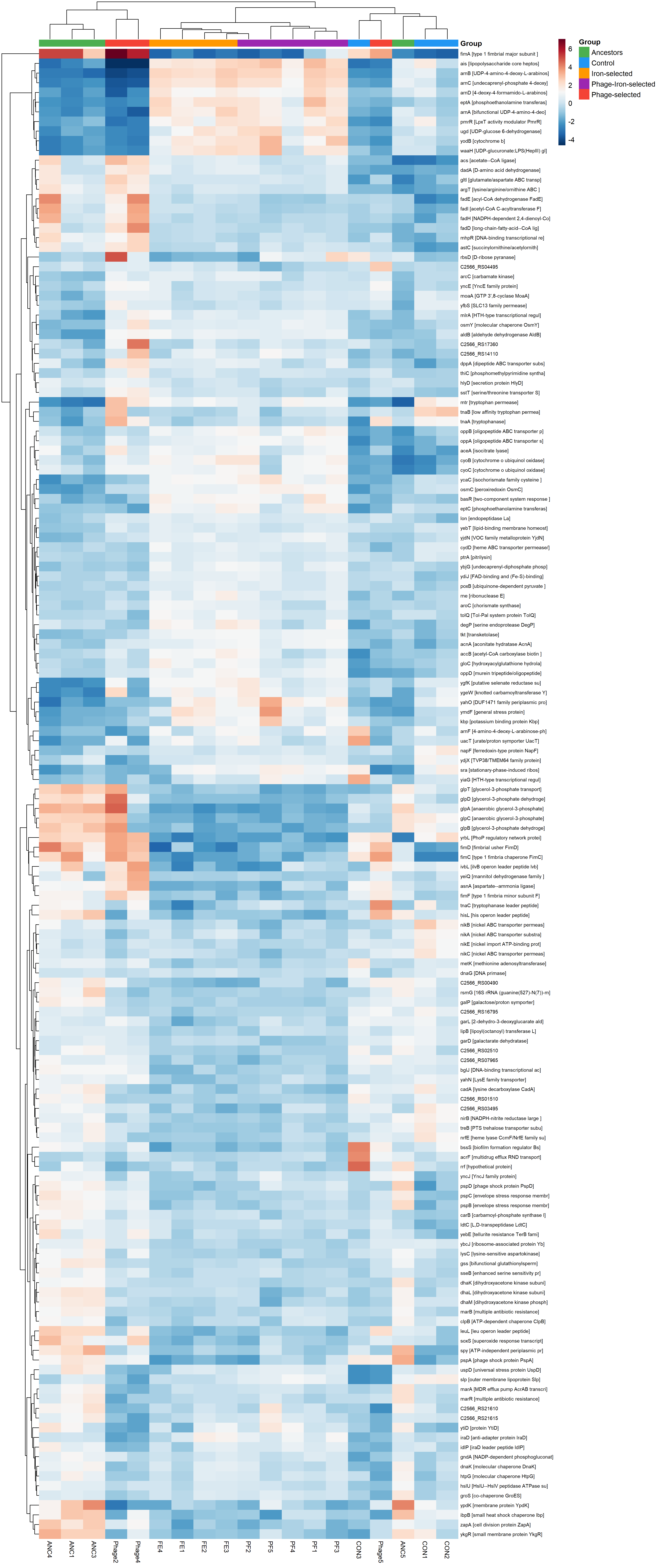

Supplement: Supplementary file 1 [file antibiotics-15-00684-s001.zip › SUPPLEMENTARY FOLDER/analysis_CON3/heatmap_top_DE_genes.png]

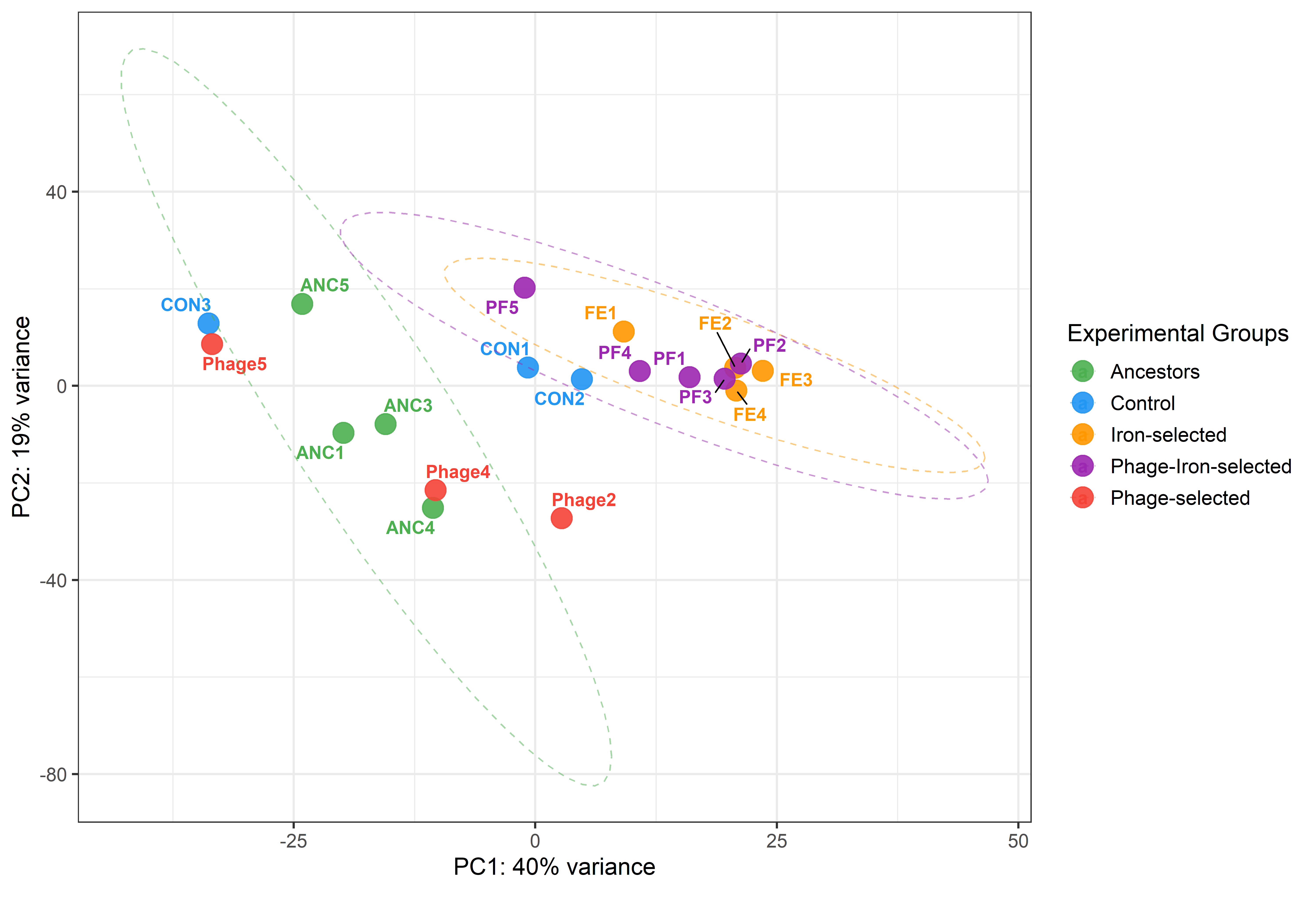

Supplement: Supplementary file 1 [file antibiotics-15-00684-s001.zip › SUPPLEMENTARY FOLDER/analysis_CON3/PCA_all_samples.png]

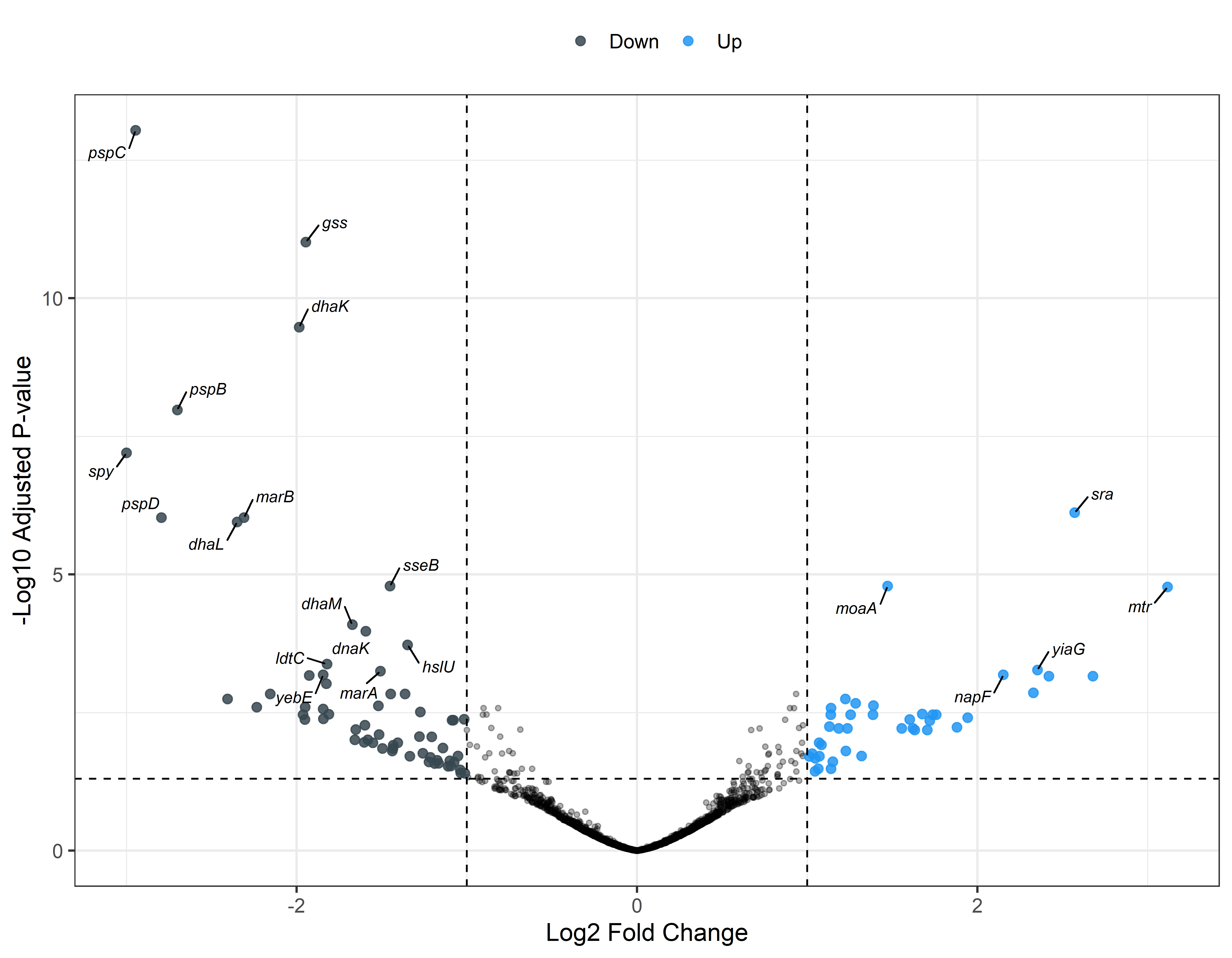

Supplement: Supplementary file 1 [file antibiotics-15-00684-s001.zip › SUPPLEMENTARY FOLDER/analysis_CON3/volcano_CON_vs_ANC.png]

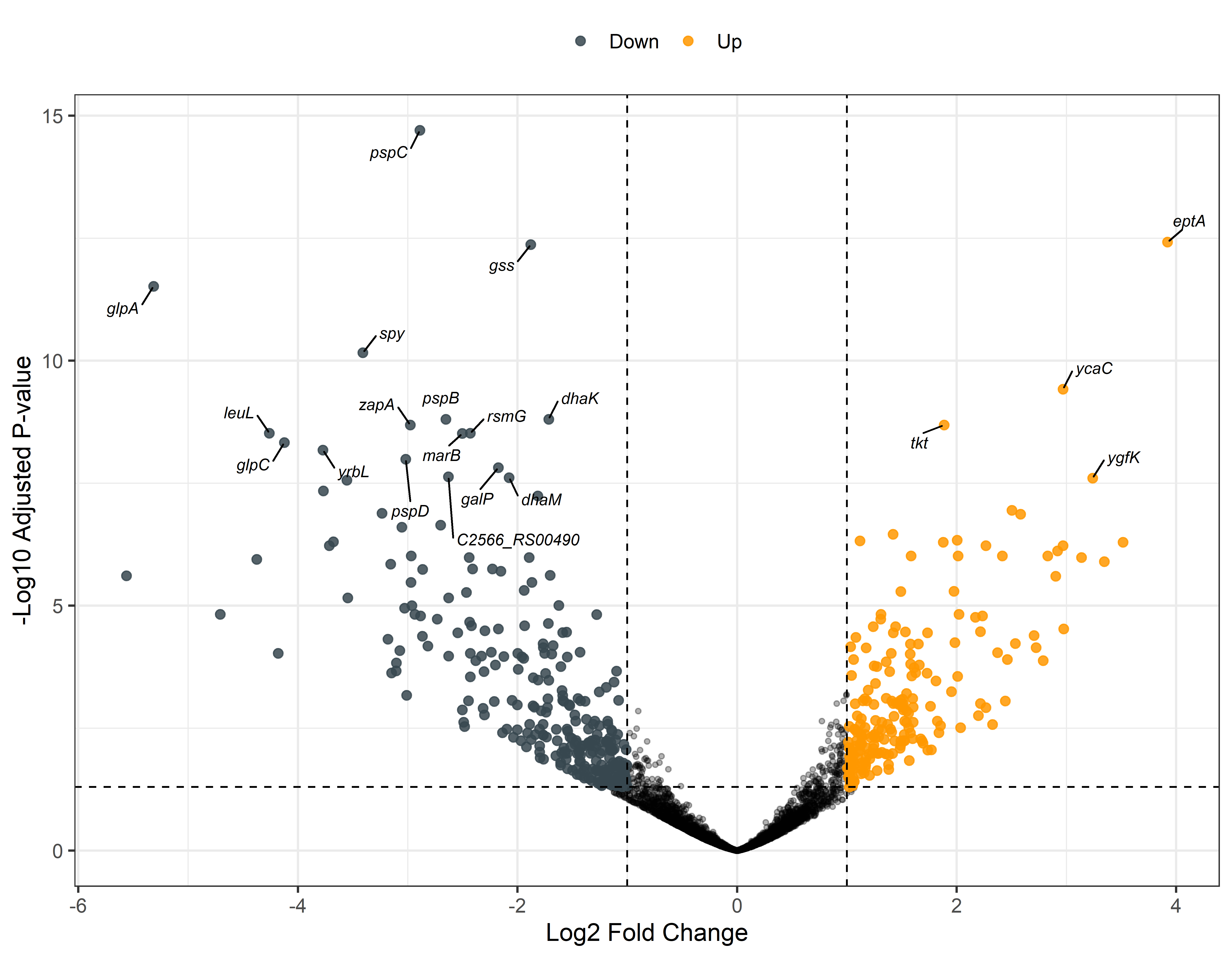

Supplement: Supplementary file 1 [file antibiotics-15-00684-s001.zip › SUPPLEMENTARY FOLDER/analysis_CON3/volcano_FE_vs_ANC.png]

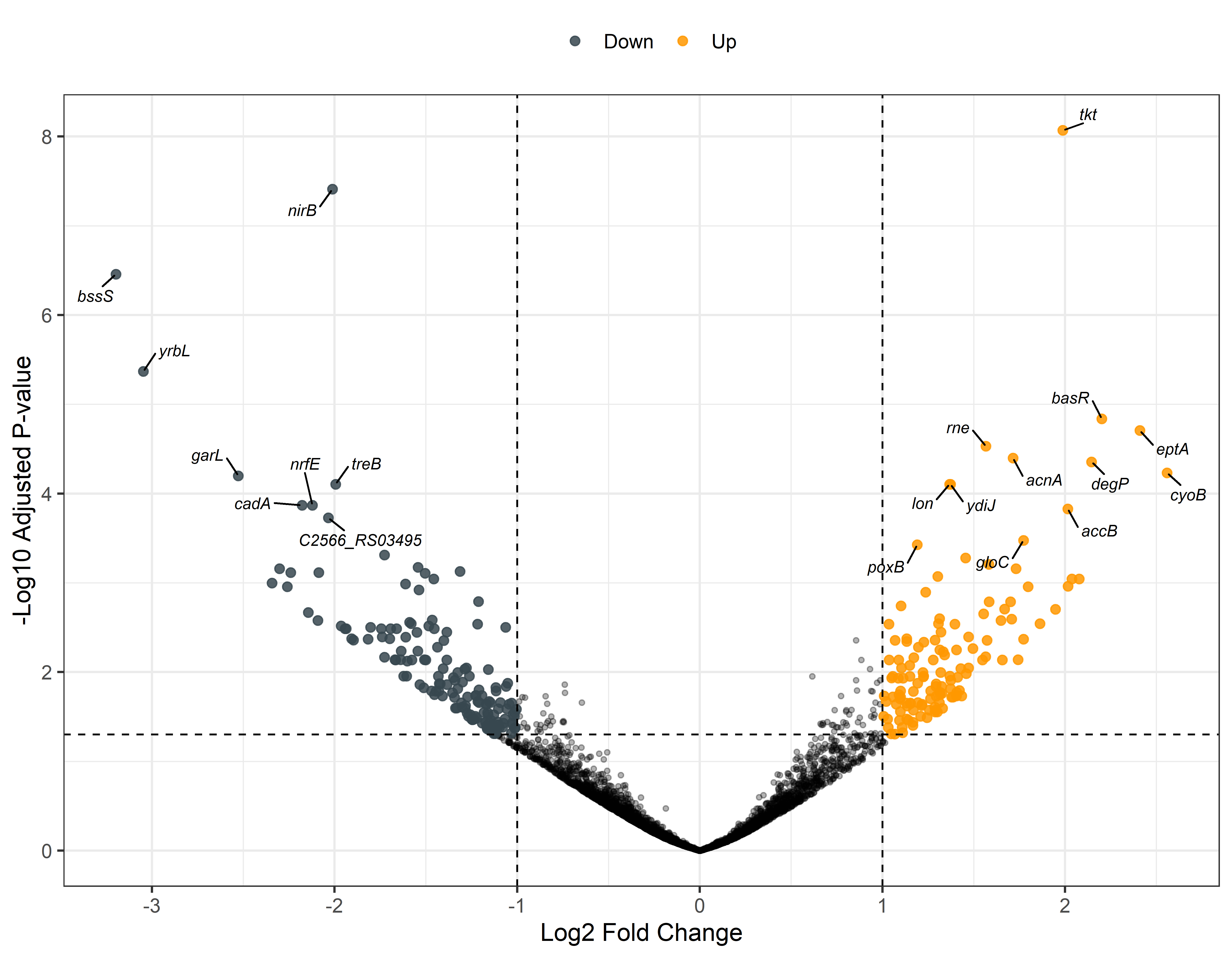

Supplement: Supplementary file 1 [file antibiotics-15-00684-s001.zip › SUPPLEMENTARY FOLDER/analysis_CON3/volcano_FE_vs_CON.png]

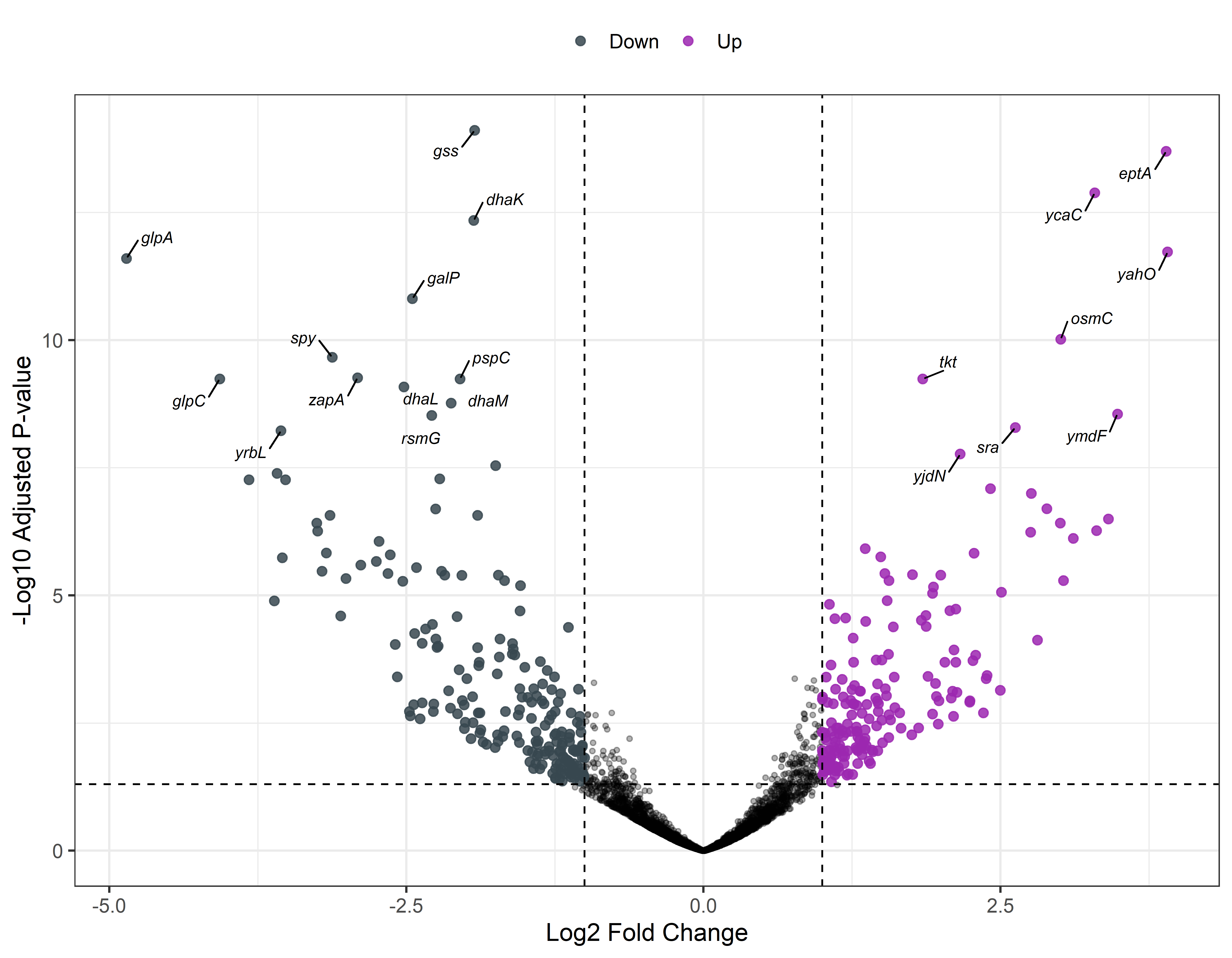

Supplement: Supplementary file 1 [file antibiotics-15-00684-s001.zip › SUPPLEMENTARY FOLDER/analysis_CON3/volcano_PF_vs_ANC.png]

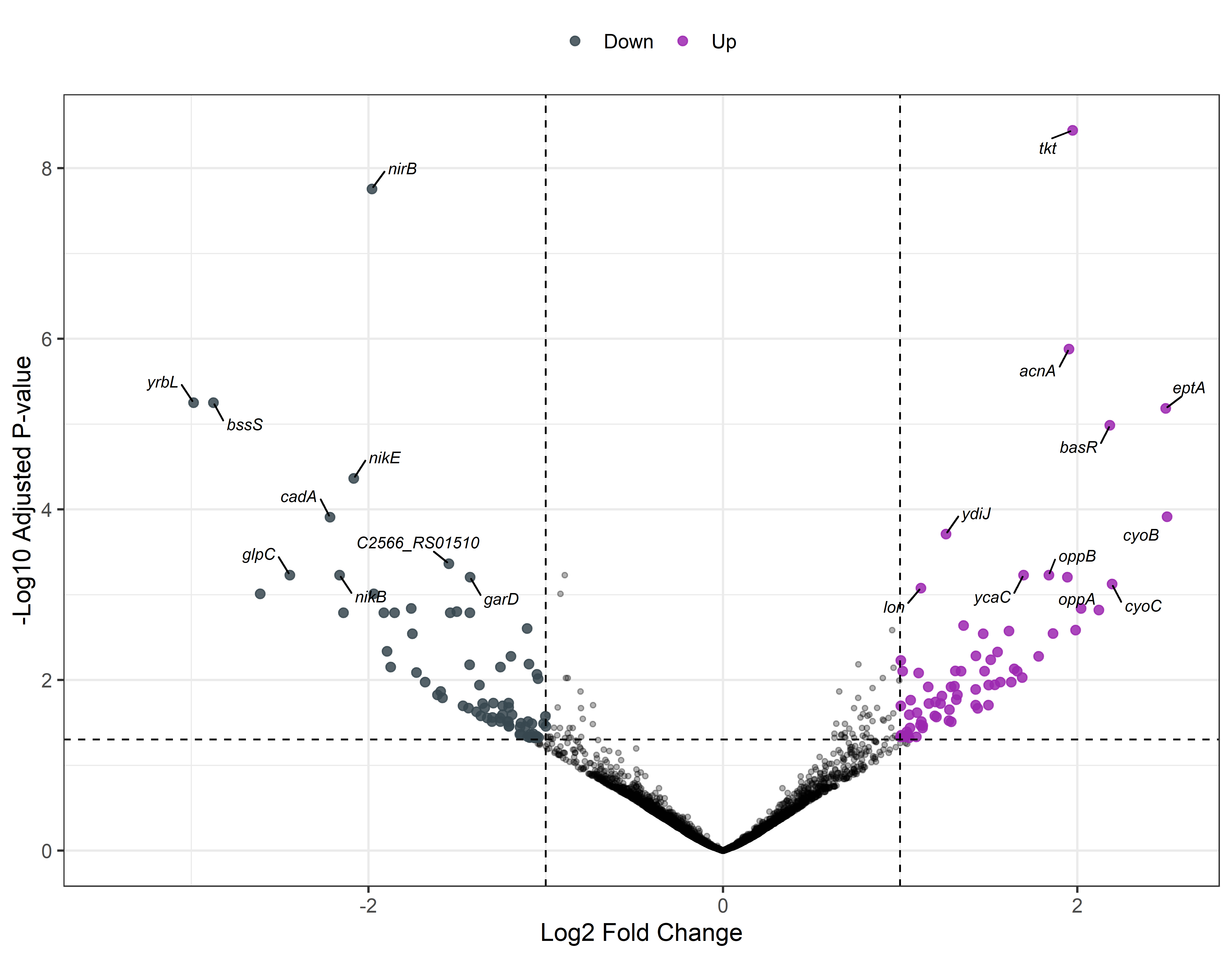

Supplement: Supplementary file 1 [file antibiotics-15-00684-s001.zip › SUPPLEMENTARY FOLDER/analysis_CON3/volcano_PF_vs_CON.png]

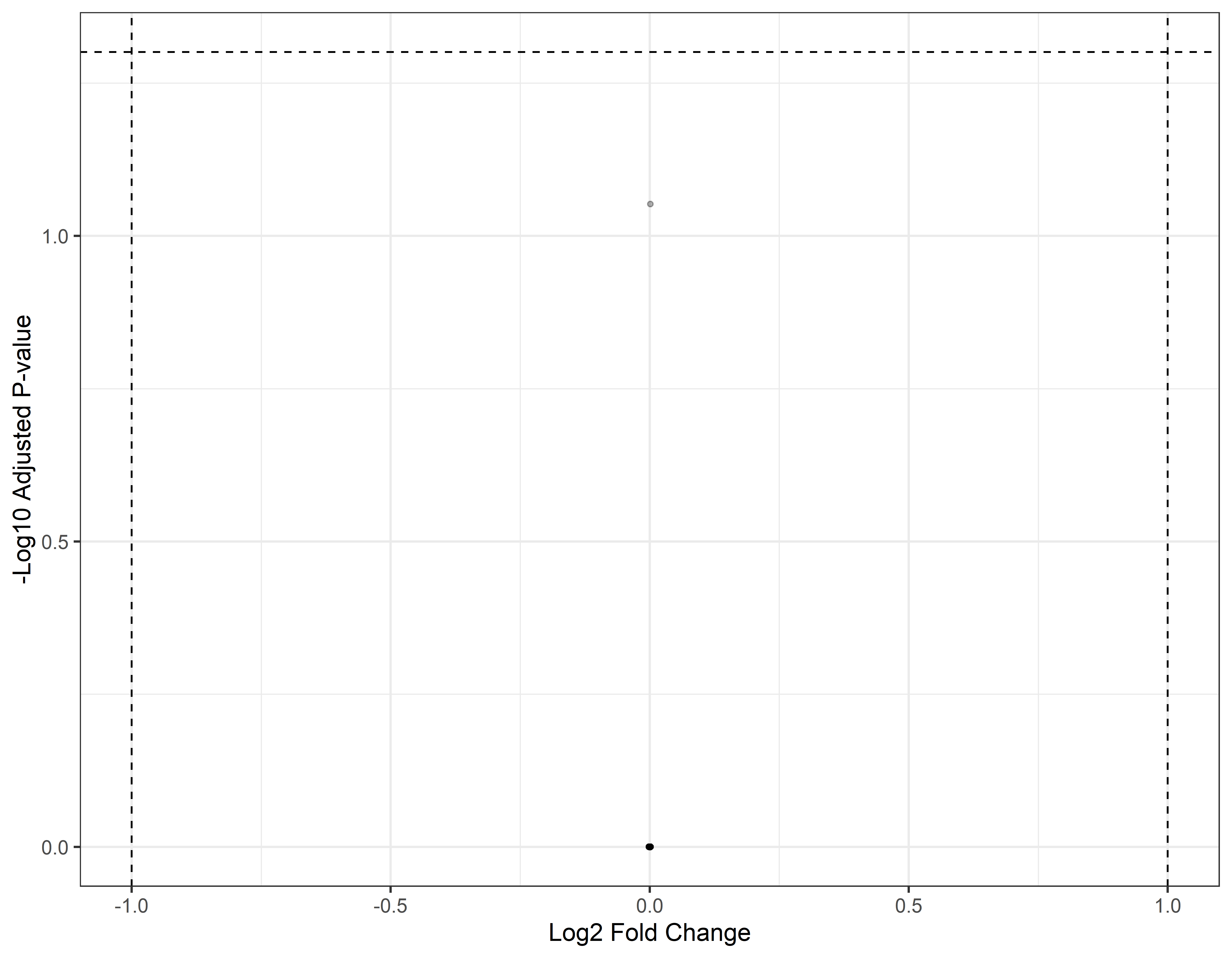

Supplement: Supplementary file 1 [file antibiotics-15-00684-s001.zip › SUPPLEMENTARY FOLDER/analysis_CON3/volcano_PF_vs_FE.png]

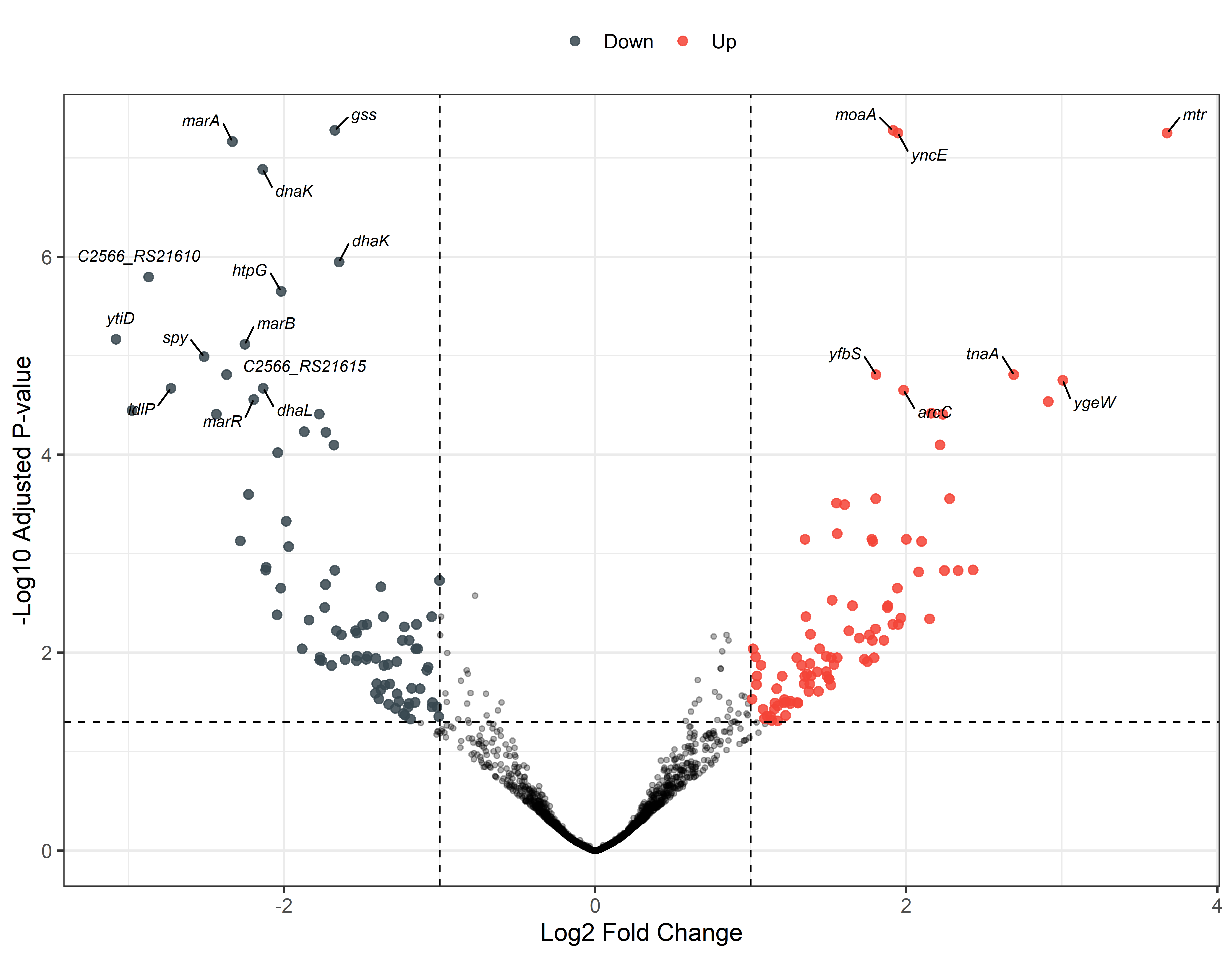

Supplement: Supplementary file 1 [file antibiotics-15-00684-s001.zip › SUPPLEMENTARY FOLDER/analysis_CON3/volcano_Phage_vs_ANC.png]

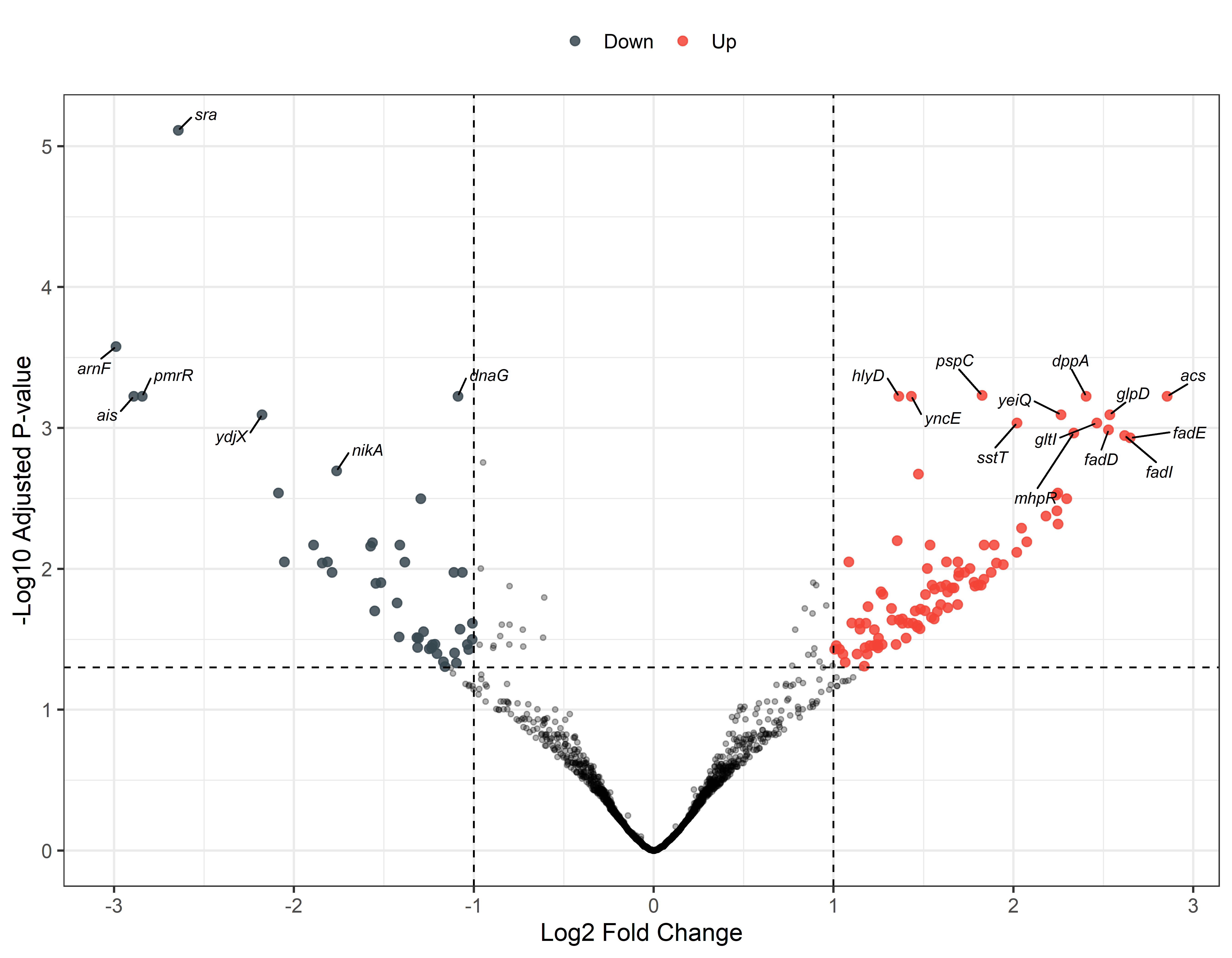

Supplement: Supplementary file 1 [file antibiotics-15-00684-s001.zip › SUPPLEMENTARY FOLDER/analysis_CON3/volcano_Phage_vs_CON.png]

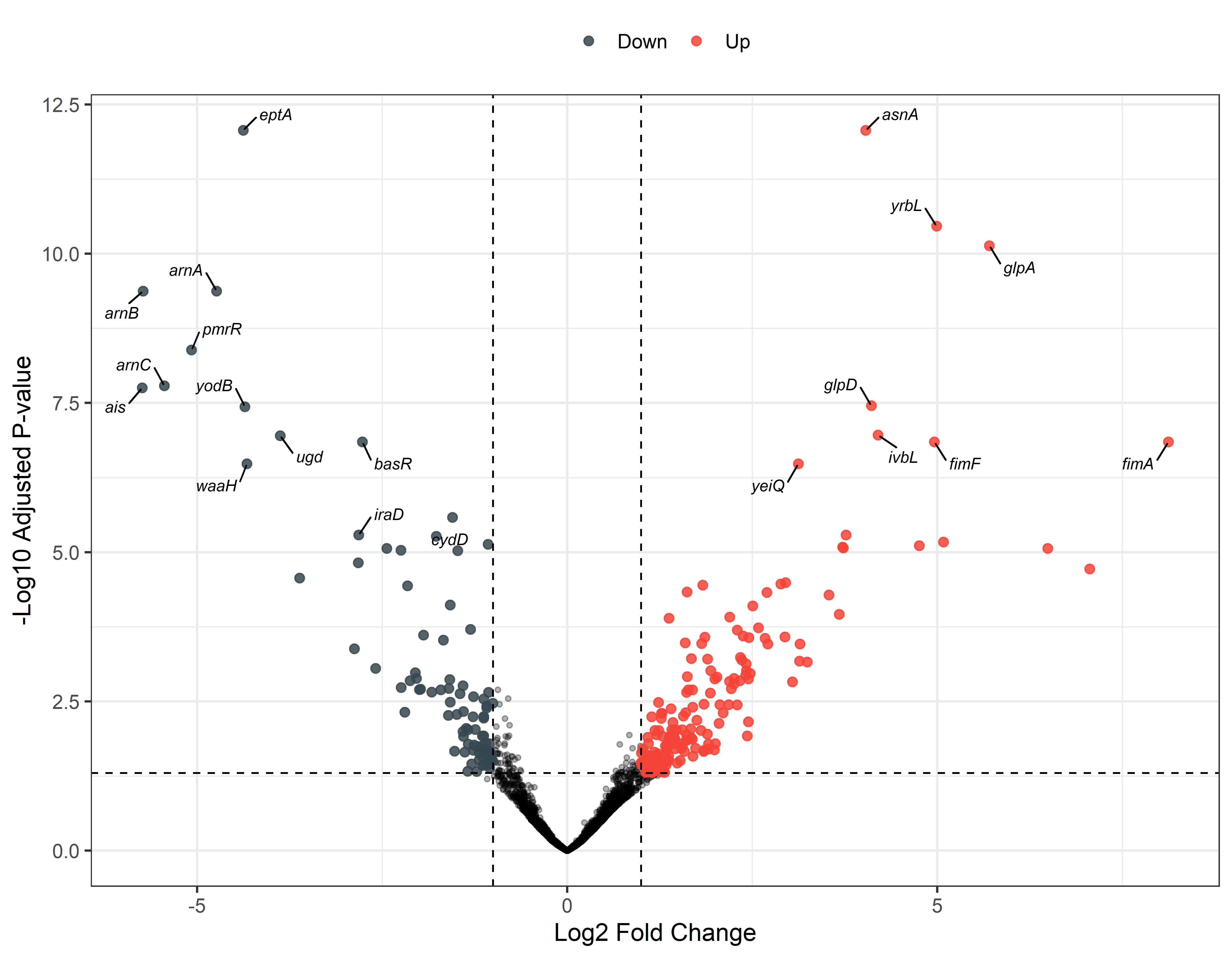

Supplement: Supplementary file 1 [file antibiotics-15-00684-s001.zip › SUPPLEMENTARY FOLDER/analysis_CON3/volcano_Phage_vs_FE.png]

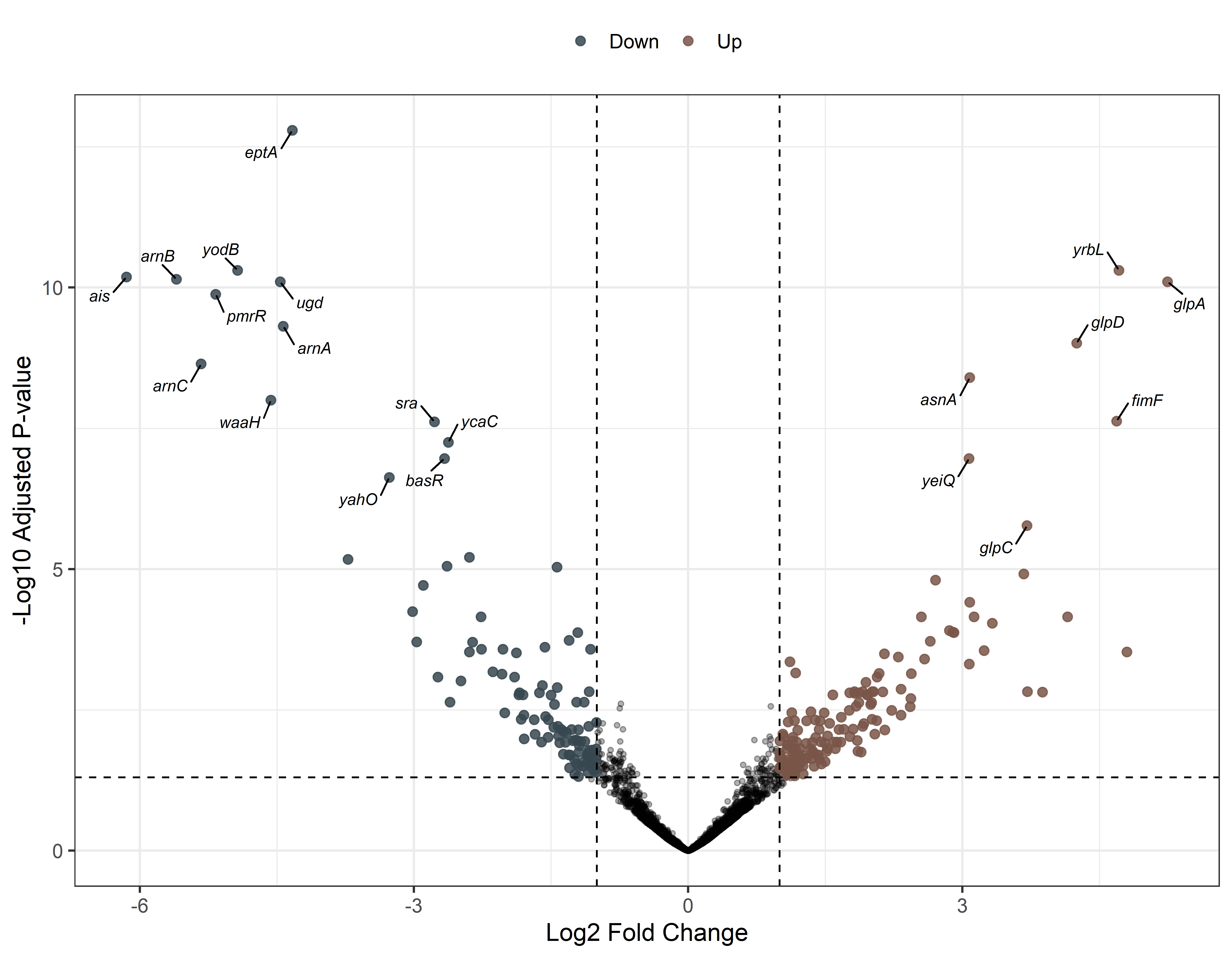

Supplement: Supplementary file 1 [file antibiotics-15-00684-s001.zip › SUPPLEMENTARY FOLDER/analysis_CON3/volcano_Phage_vs_PF.png]

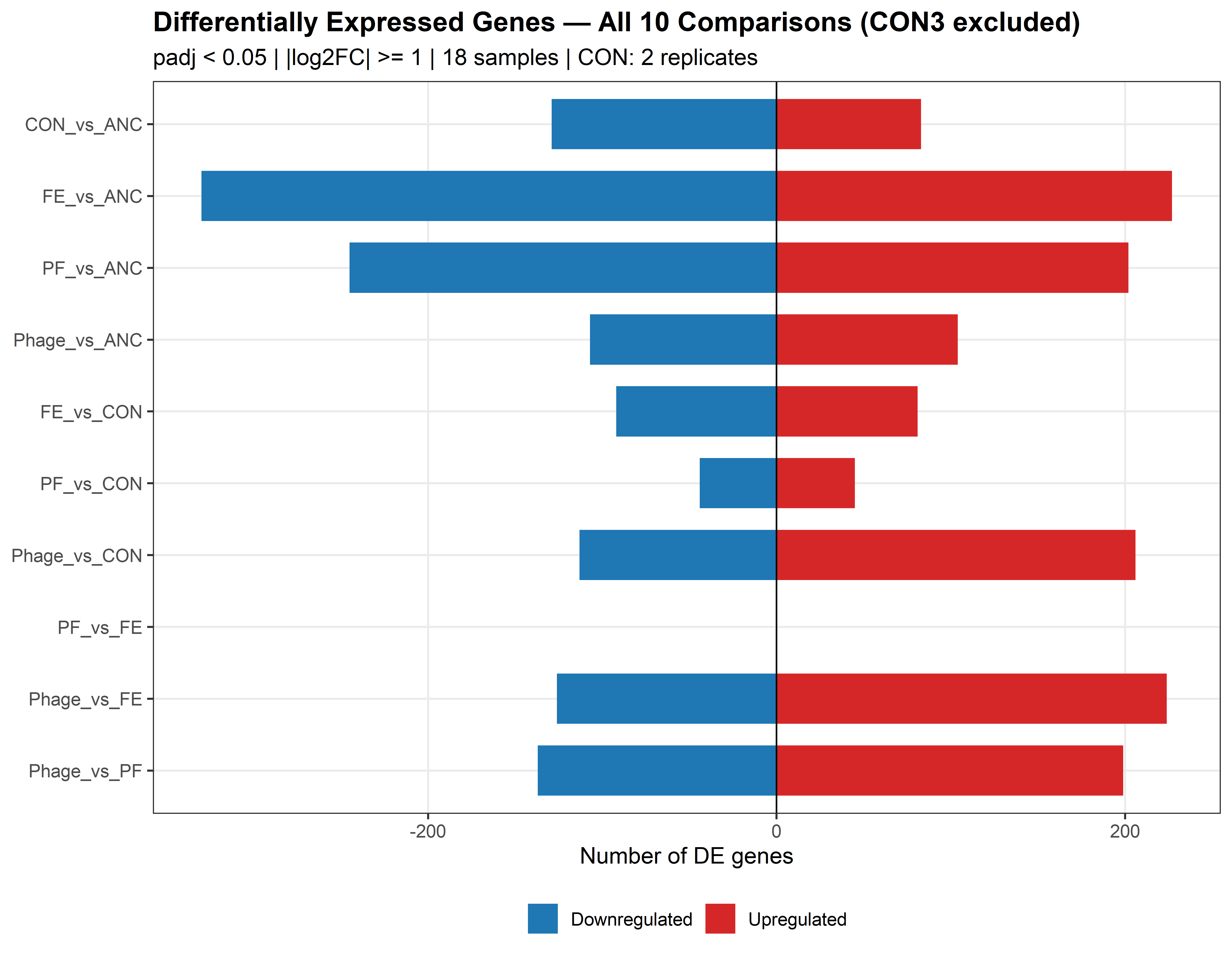

Supplement: Supplementary file 1 [file antibiotics-15-00684-s001.zip › SUPPLEMENTARY FOLDER/analysis_noCON3/DE_gene_counts_barplot_noCON3.png]

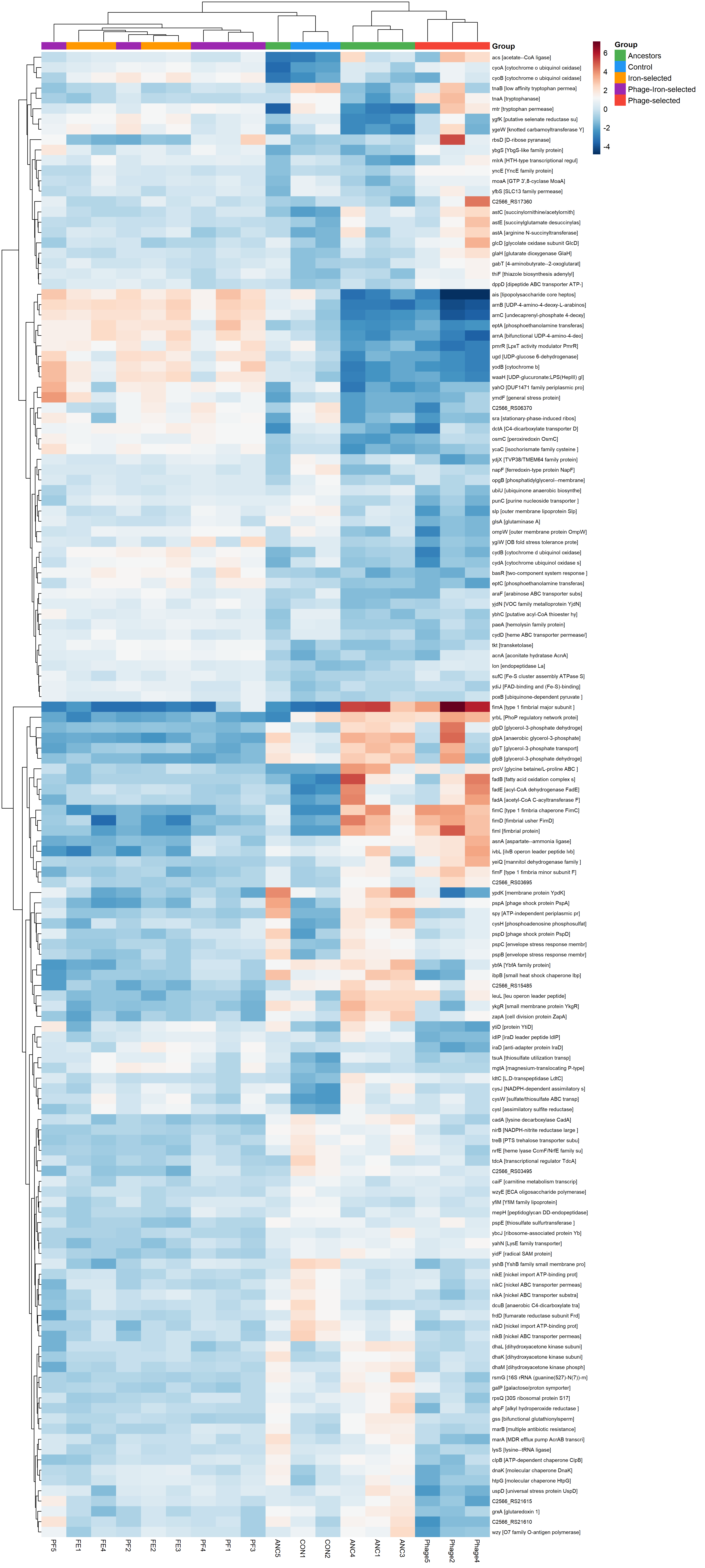

Supplement: Supplementary file 1 [file antibiotics-15-00684-s001.zip › SUPPLEMENTARY FOLDER/analysis_noCON3/heatmap_top_DE_genes_noCON3.png]

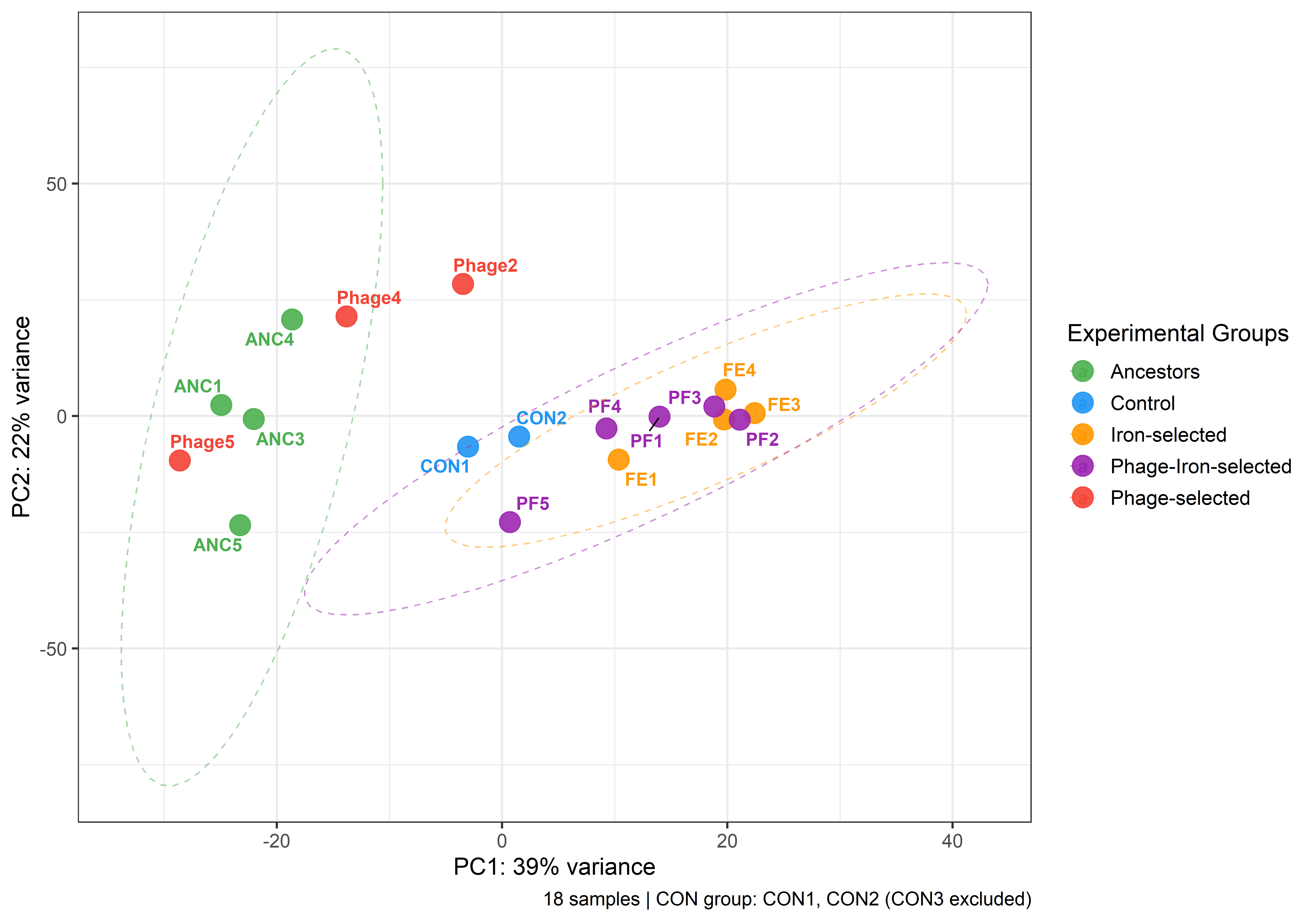

Supplement: Supplementary file 1 [file antibiotics-15-00684-s001.zip › SUPPLEMENTARY FOLDER/analysis_noCON3/PCA_all_samples_noCON3.png]

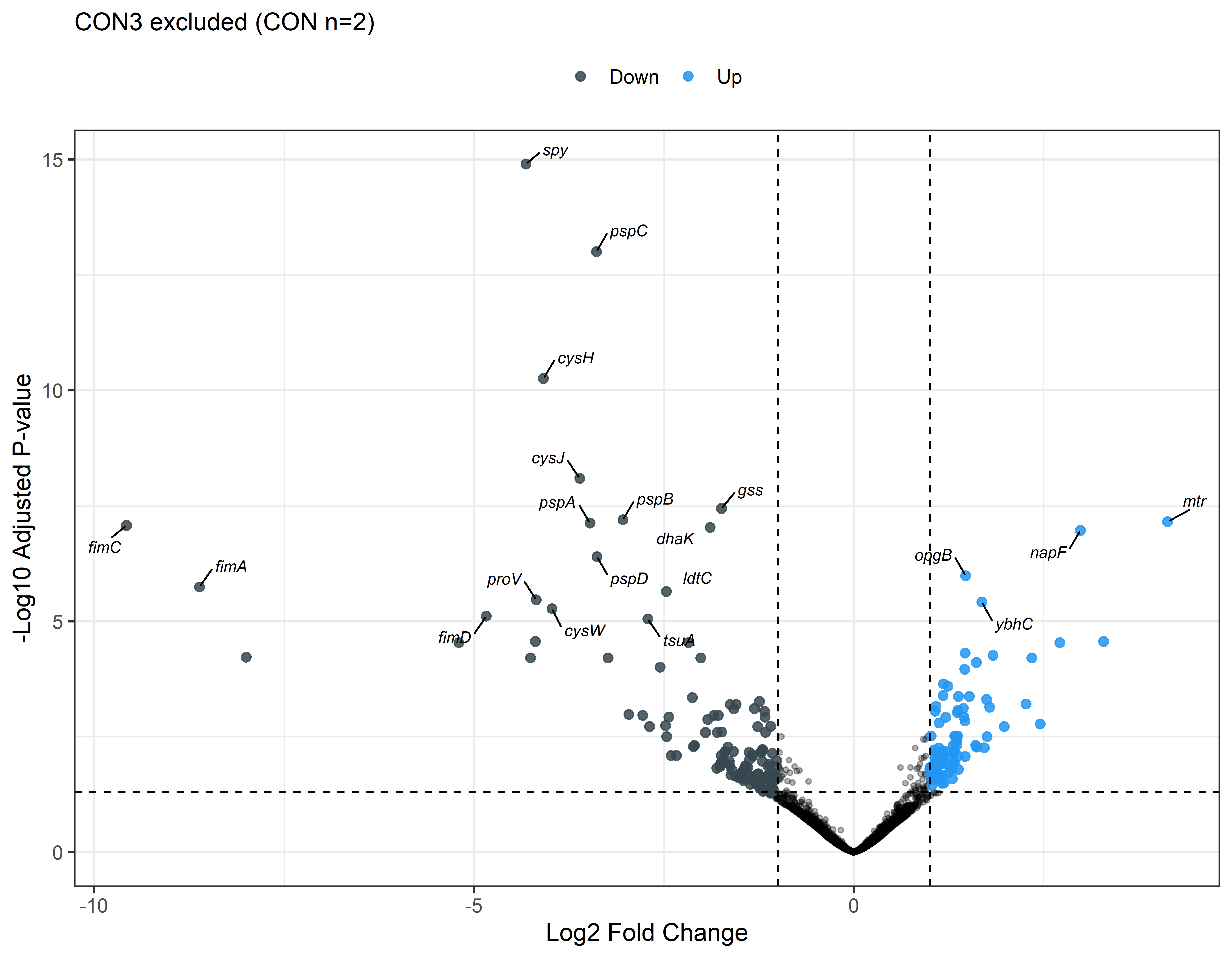

Supplement: Supplementary file 1 [file antibiotics-15-00684-s001.zip › SUPPLEMENTARY FOLDER/analysis_noCON3/volcano_CON_vs_ANC_noCON3.png]

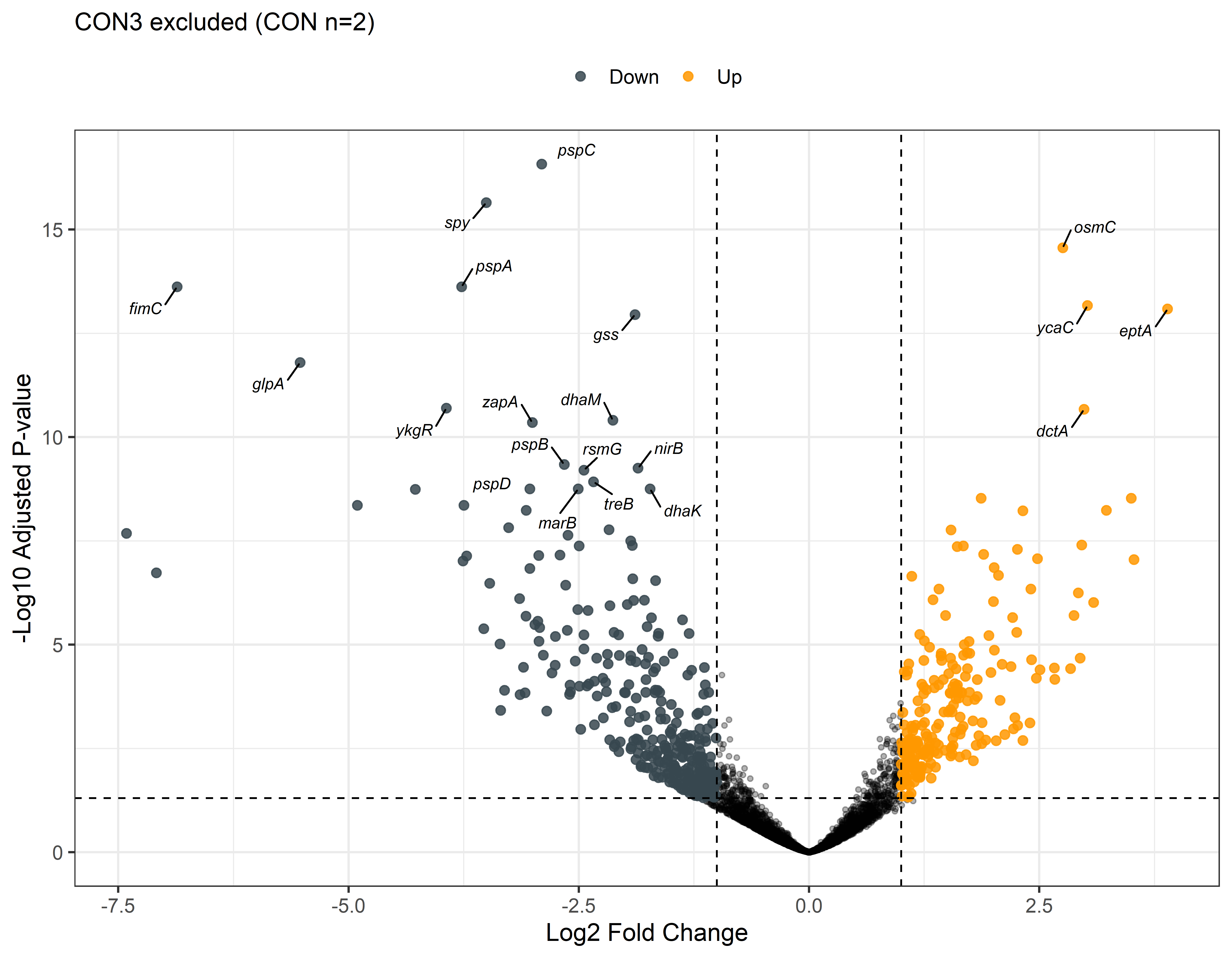

Supplement: Supplementary file 1 [file antibiotics-15-00684-s001.zip › SUPPLEMENTARY FOLDER/analysis_noCON3/volcano_FE_vs_ANC_noCON3.png]

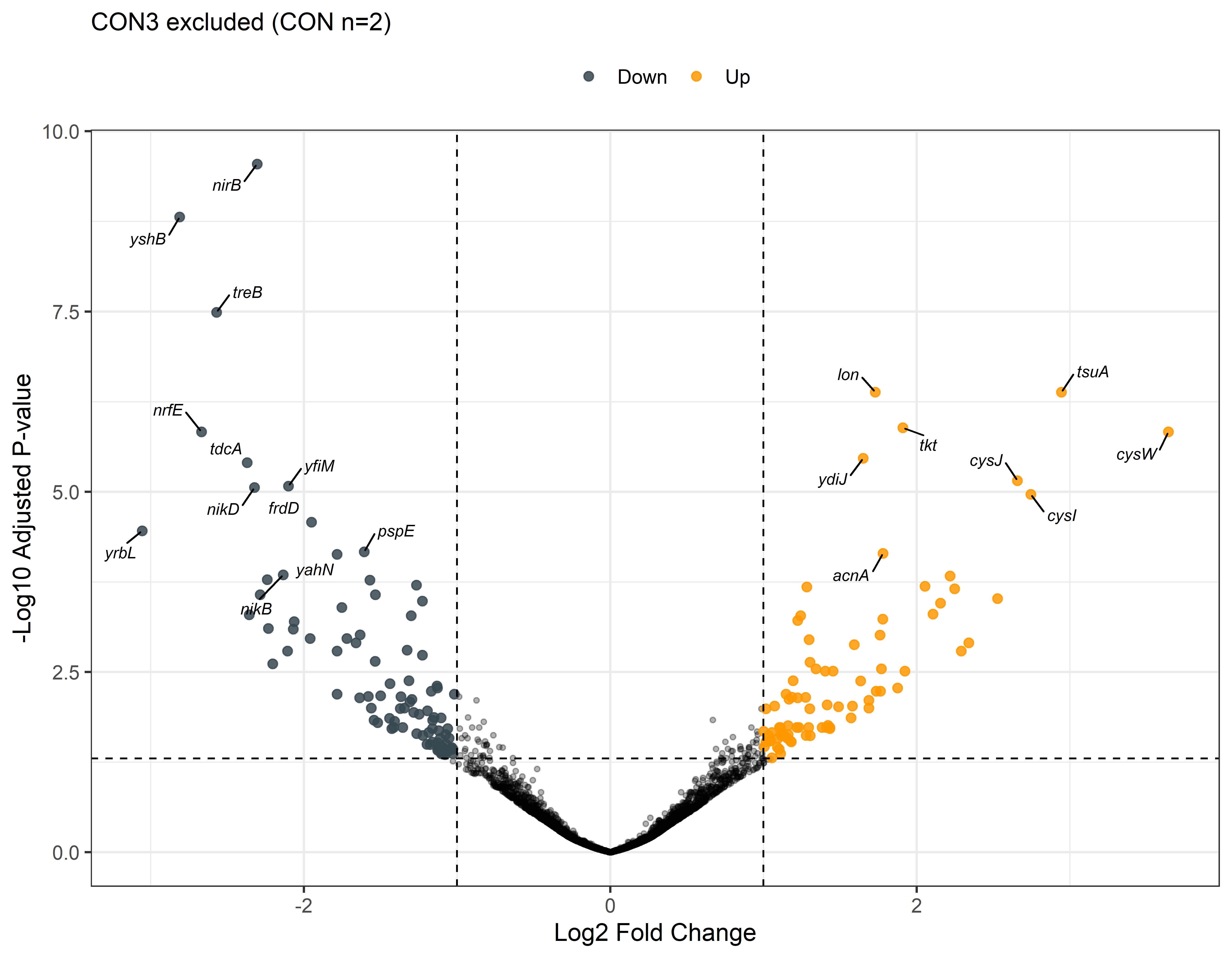

Supplement: Supplementary file 1 [file antibiotics-15-00684-s001.zip › SUPPLEMENTARY FOLDER/analysis_noCON3/volcano_FE_vs_CON_noCON3.png]

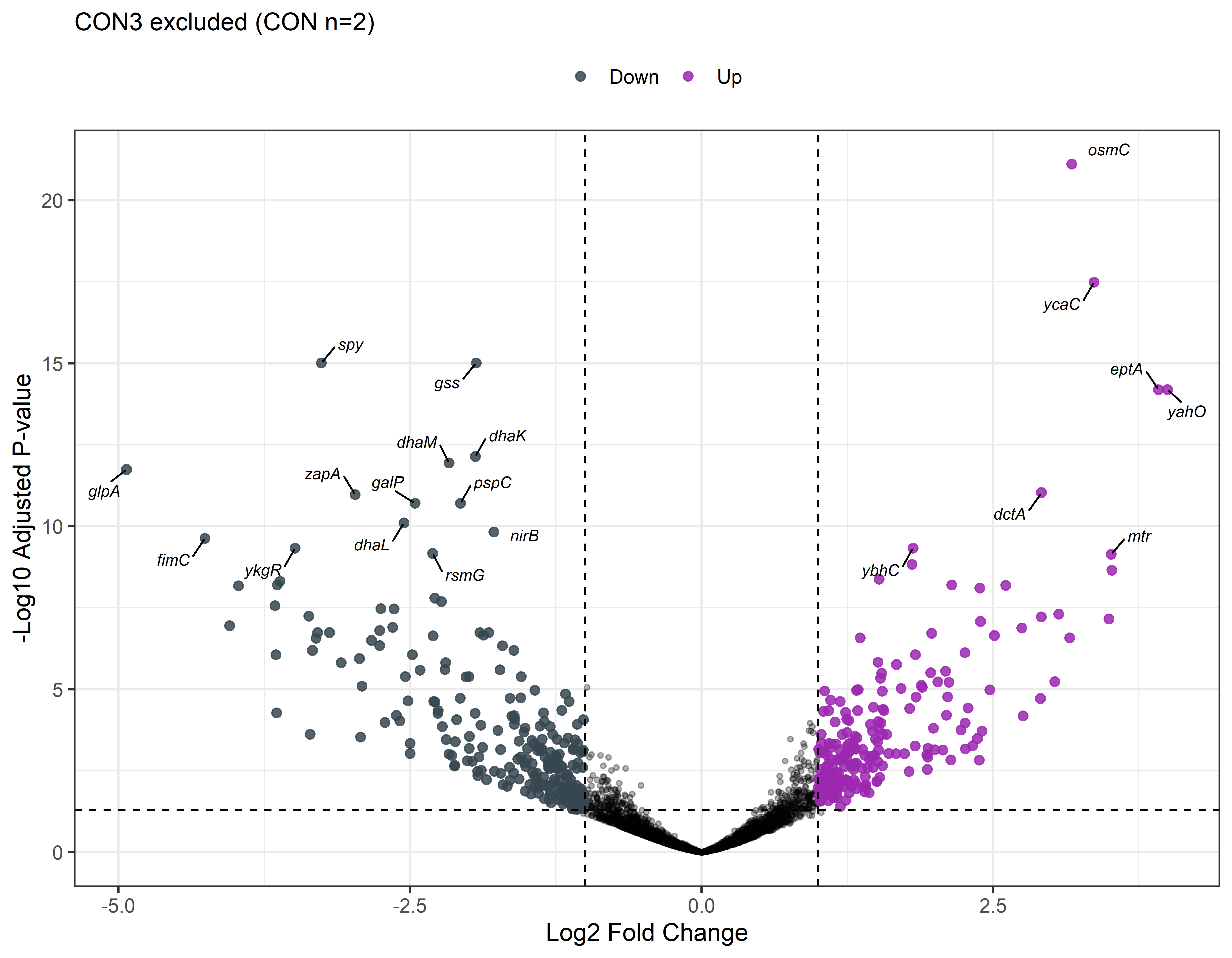

Supplement: Supplementary file 1 [file antibiotics-15-00684-s001.zip › SUPPLEMENTARY FOLDER/analysis_noCON3/volcano_PF_vs_ANC_noCON3.png]

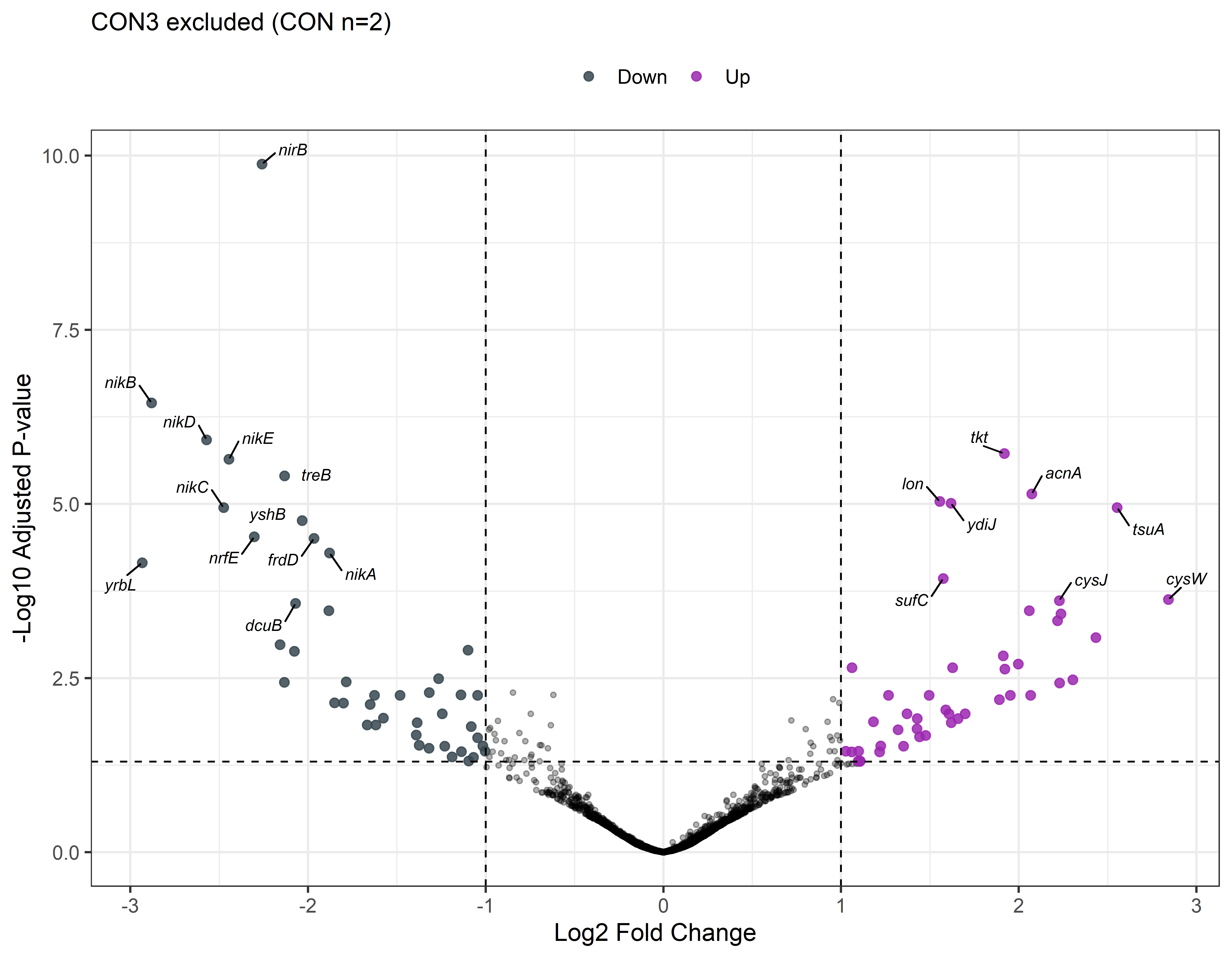

Supplement: Supplementary file 1 [file antibiotics-15-00684-s001.zip › SUPPLEMENTARY FOLDER/analysis_noCON3/volcano_PF_vs_CON_noCON3.png]

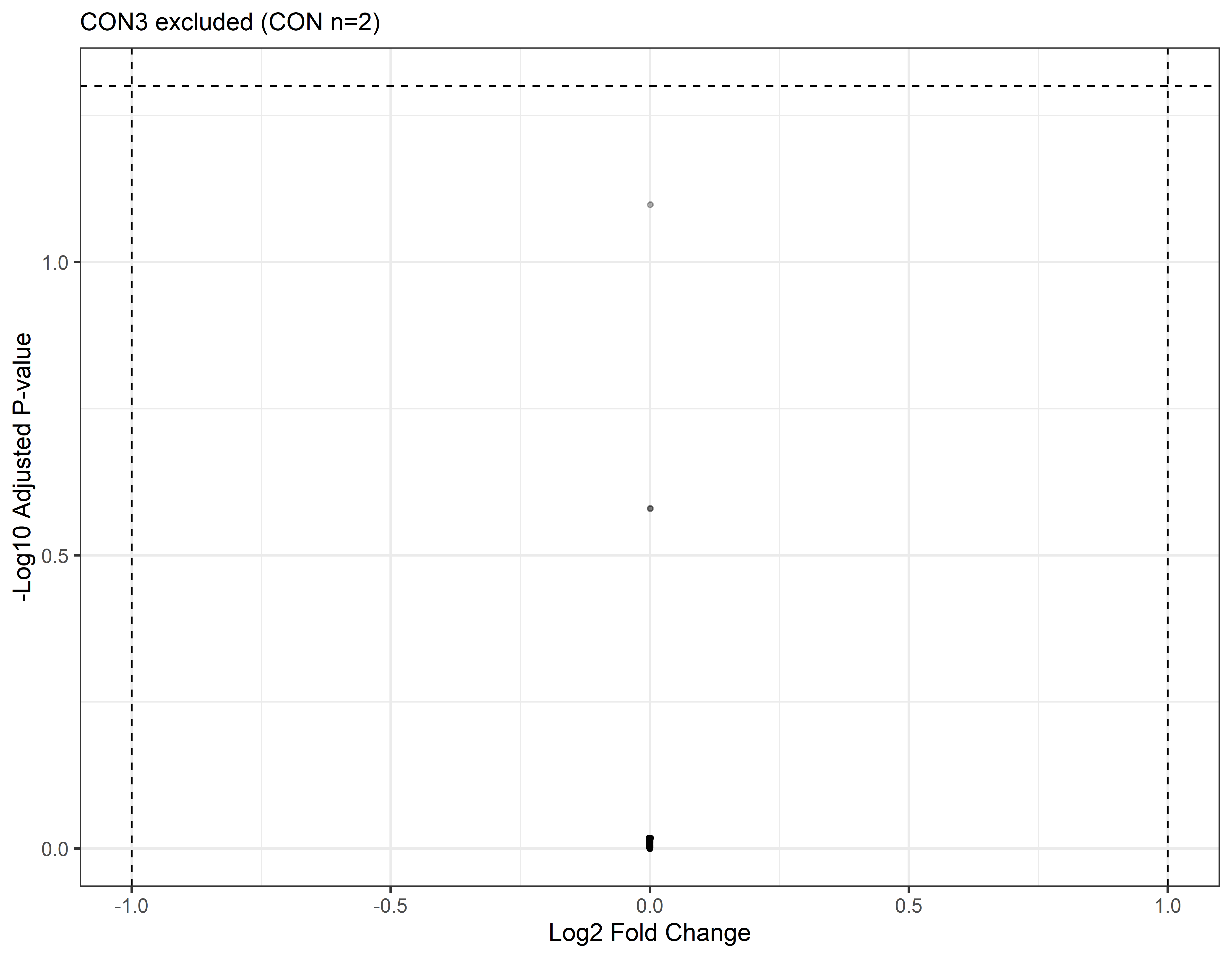

Supplement: Supplementary file 1 [file antibiotics-15-00684-s001.zip › SUPPLEMENTARY FOLDER/analysis_noCON3/volcano_PF_vs_FE_noCON3.png]

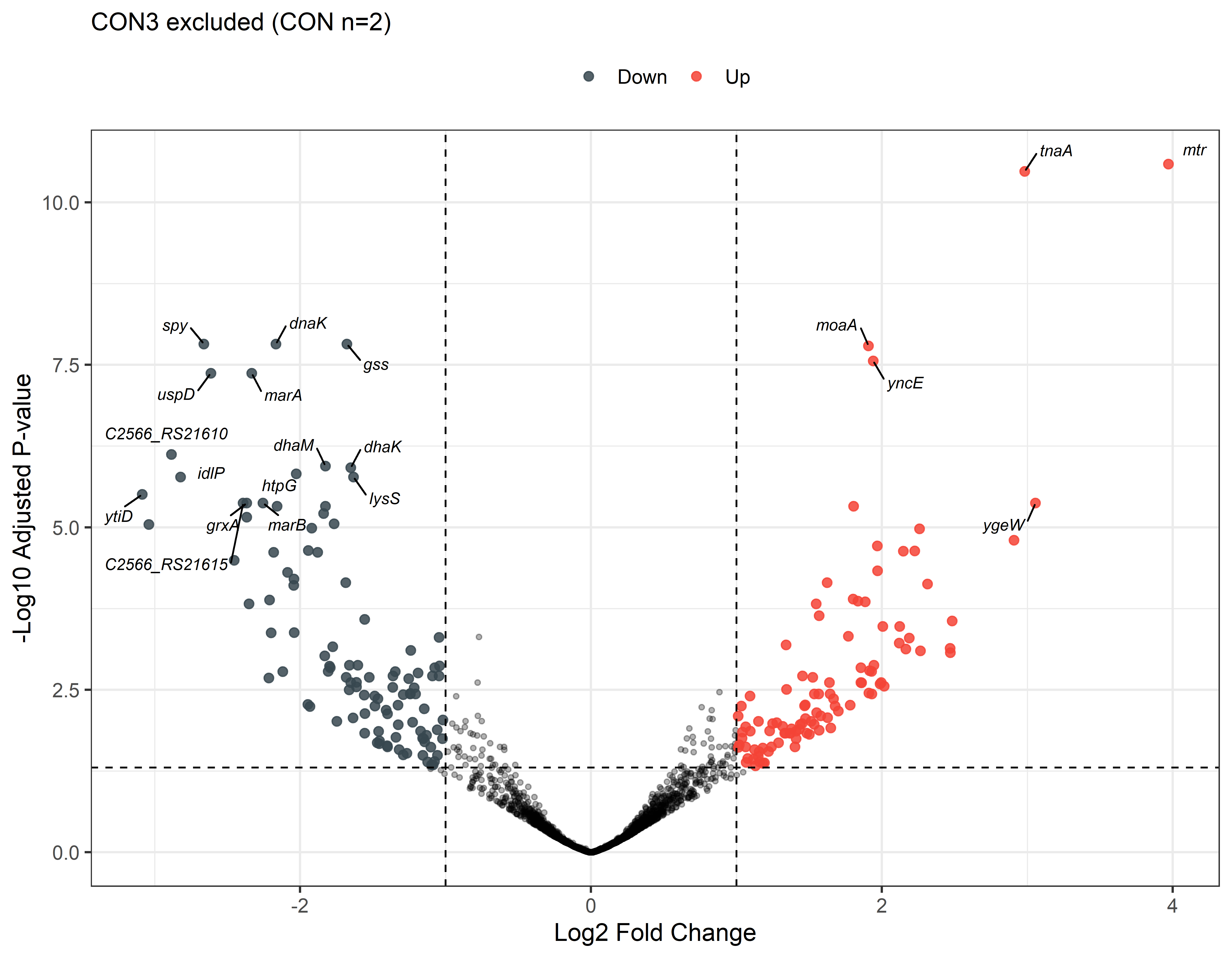

Supplement: Supplementary file 1 [file antibiotics-15-00684-s001.zip › SUPPLEMENTARY FOLDER/analysis_noCON3/volcano_Phage_vs_ANC_noCON3.png]

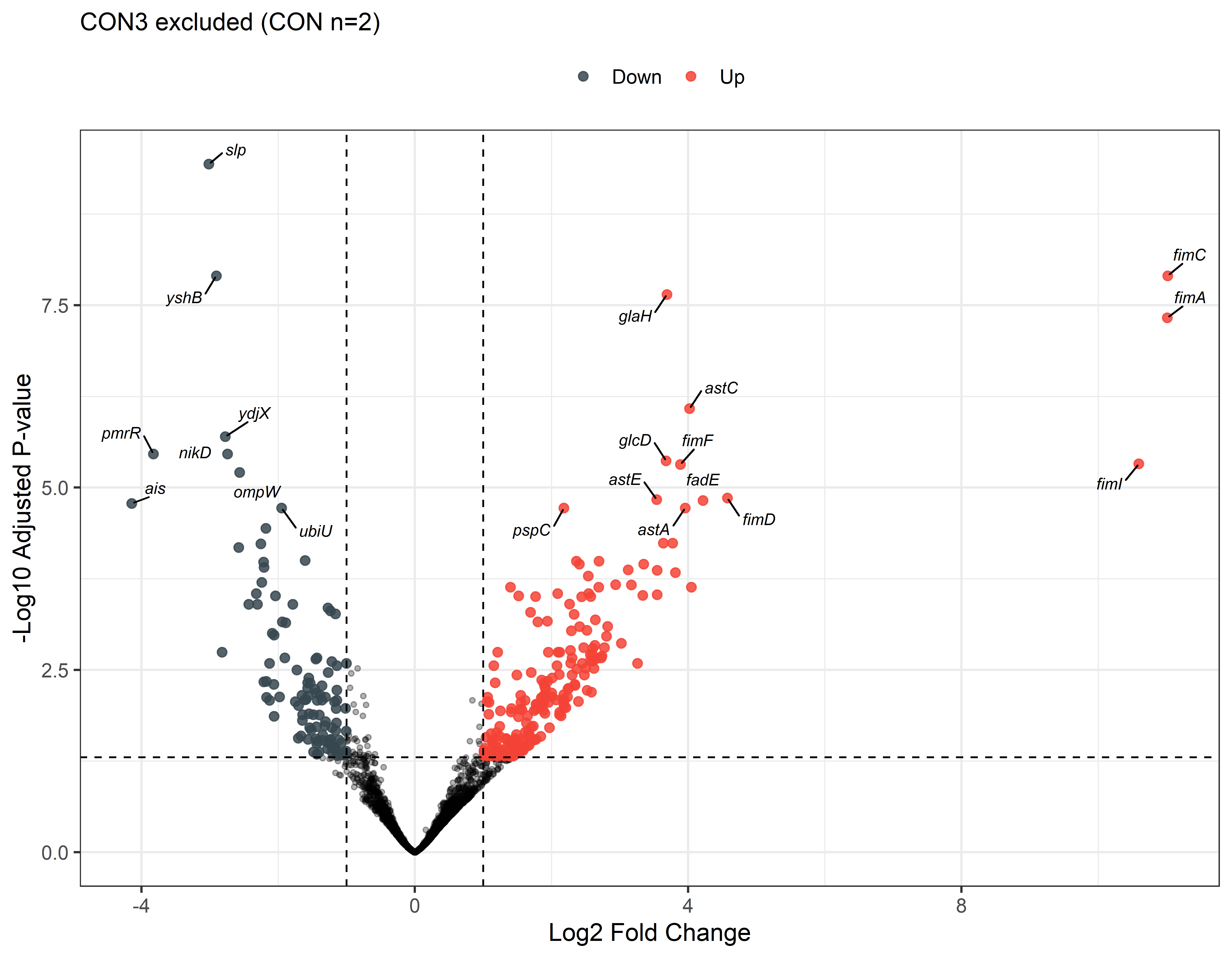

Supplement: Supplementary file 1 [file antibiotics-15-00684-s001.zip › SUPPLEMENTARY FOLDER/analysis_noCON3/volcano_Phage_vs_CON_noCON3.png]

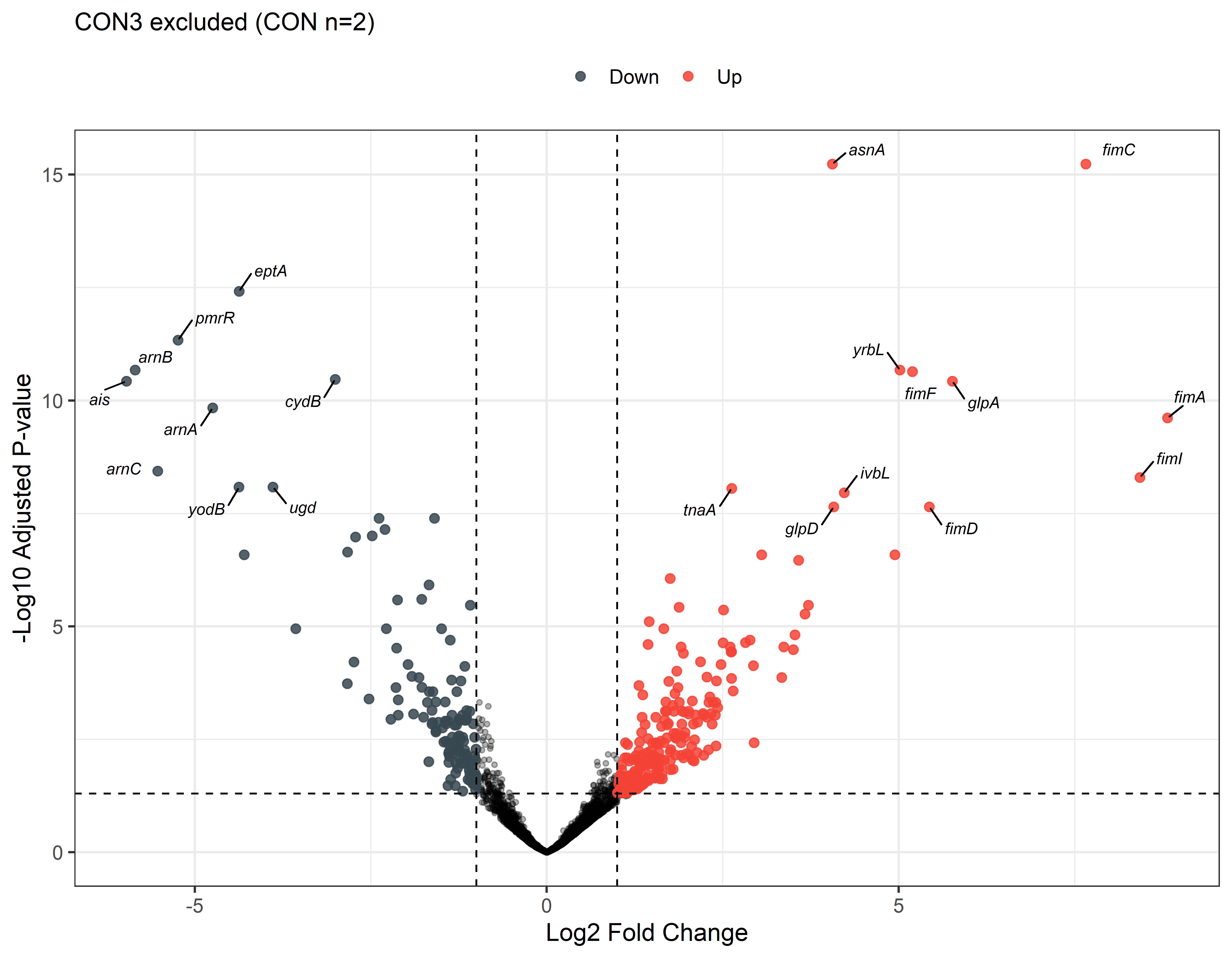

Supplement: Supplementary file 1 [file antibiotics-15-00684-s001.zip › SUPPLEMENTARY FOLDER/analysis_noCON3/volcano_Phage_vs_FE_noCON3.png]

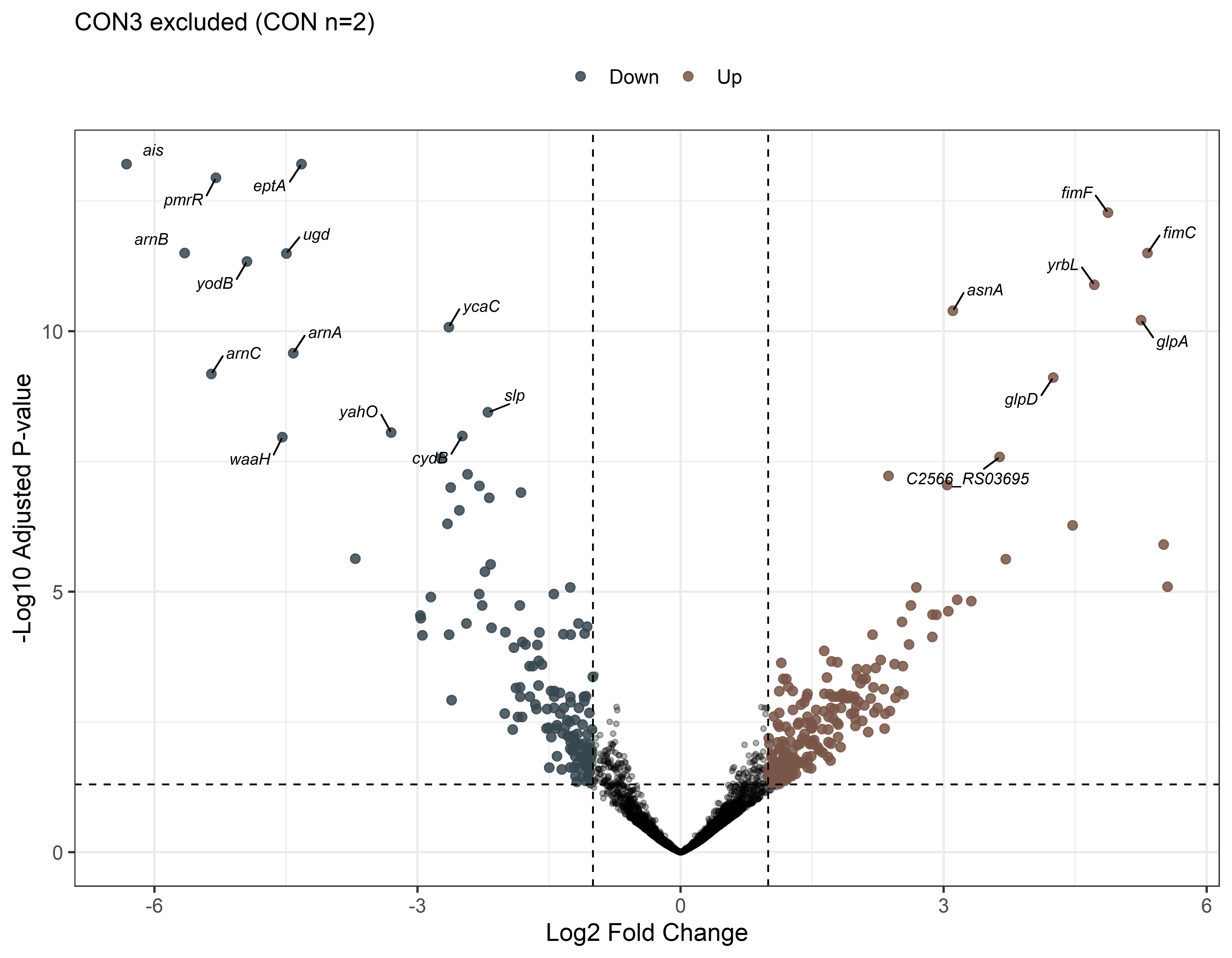

Supplement: Supplementary file 1 [file antibiotics-15-00684-s001.zip › SUPPLEMENTARY FOLDER/analysis_noCON3/volcano_Phage_vs_PF_noCON3.png]

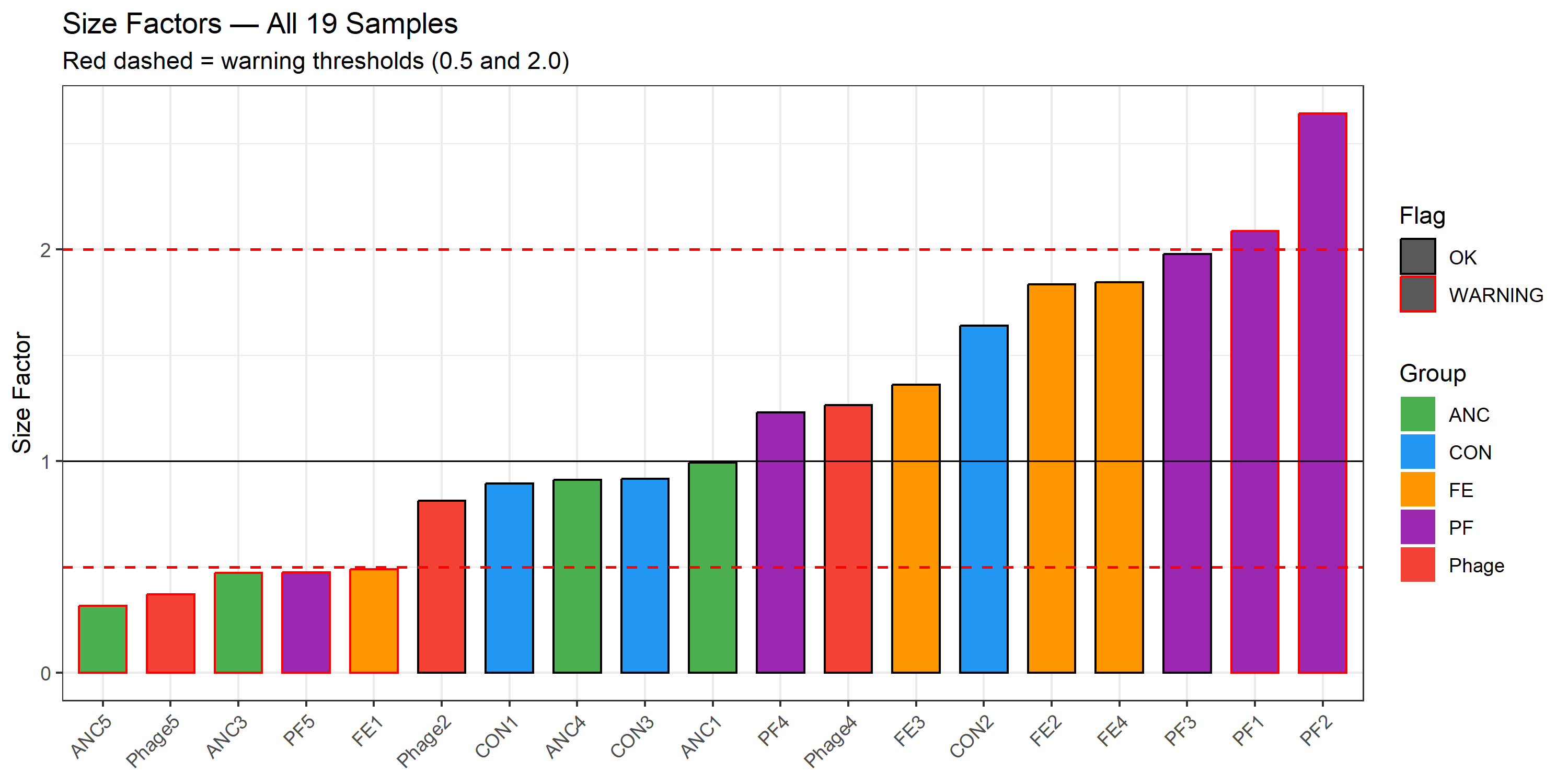

Supplement: Supplementary file 1 [file antibiotics-15-00684-s001.zip › SUPPLEMENTARY FOLDER/Sensitivity CON3/01_size_factors.png]

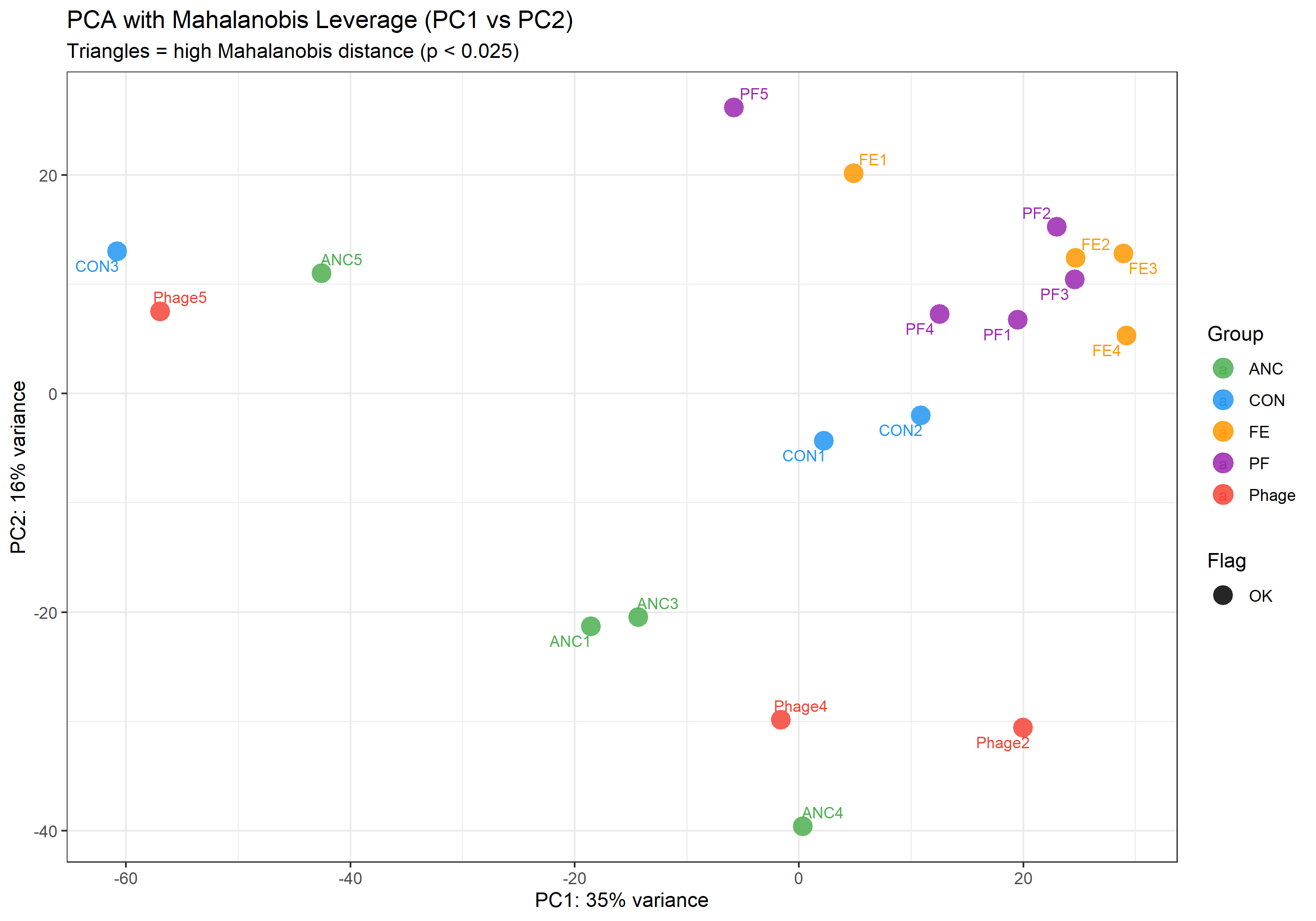

Supplement: Supplementary file 1 [file antibiotics-15-00684-s001.zip › SUPPLEMENTARY FOLDER/Sensitivity CON3/04_pca_leverage.png]

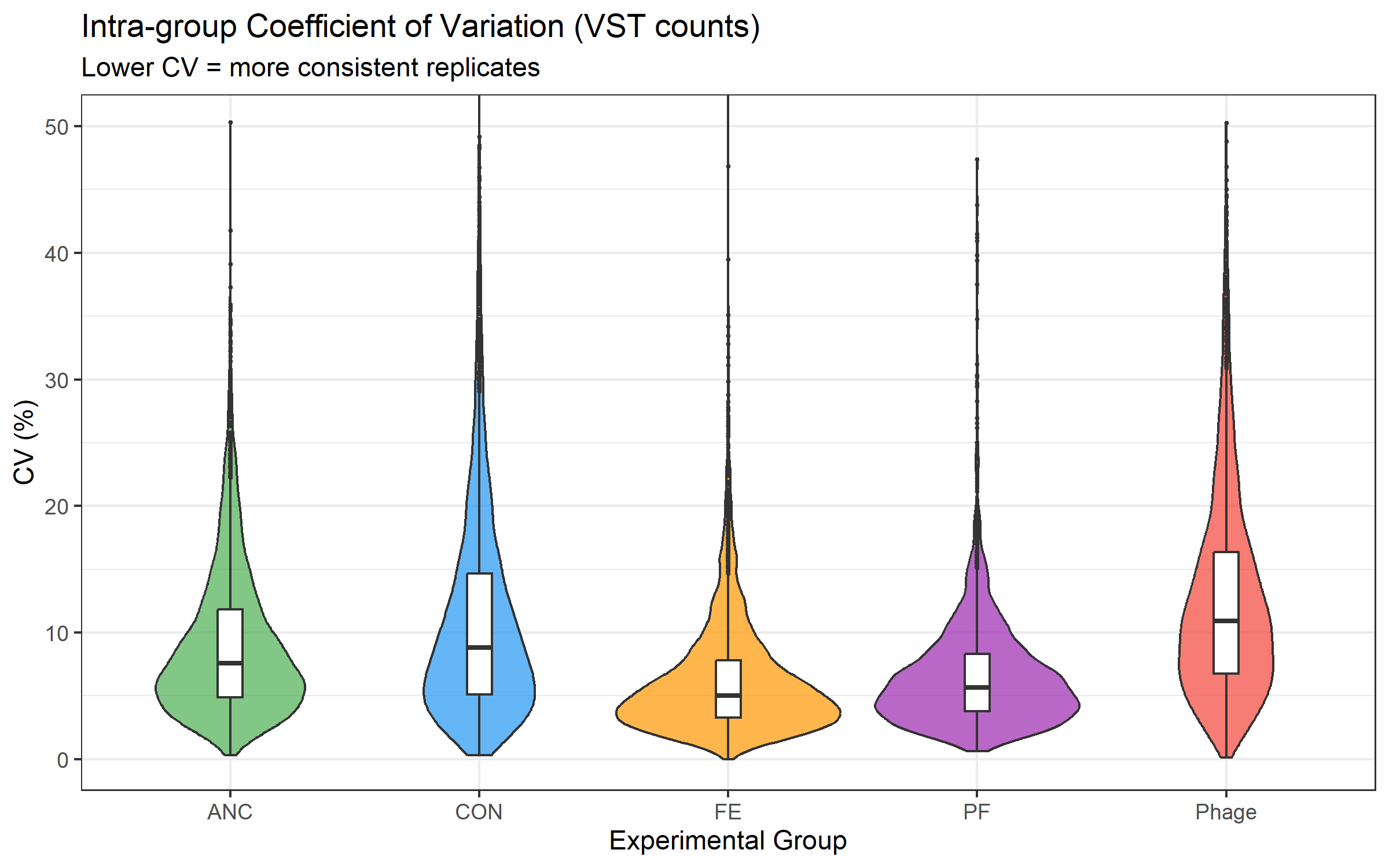

Supplement: Supplementary file 1 [file antibiotics-15-00684-s001.zip › SUPPLEMENTARY FOLDER/Sensitivity CON3/05_replicate_CV.png]

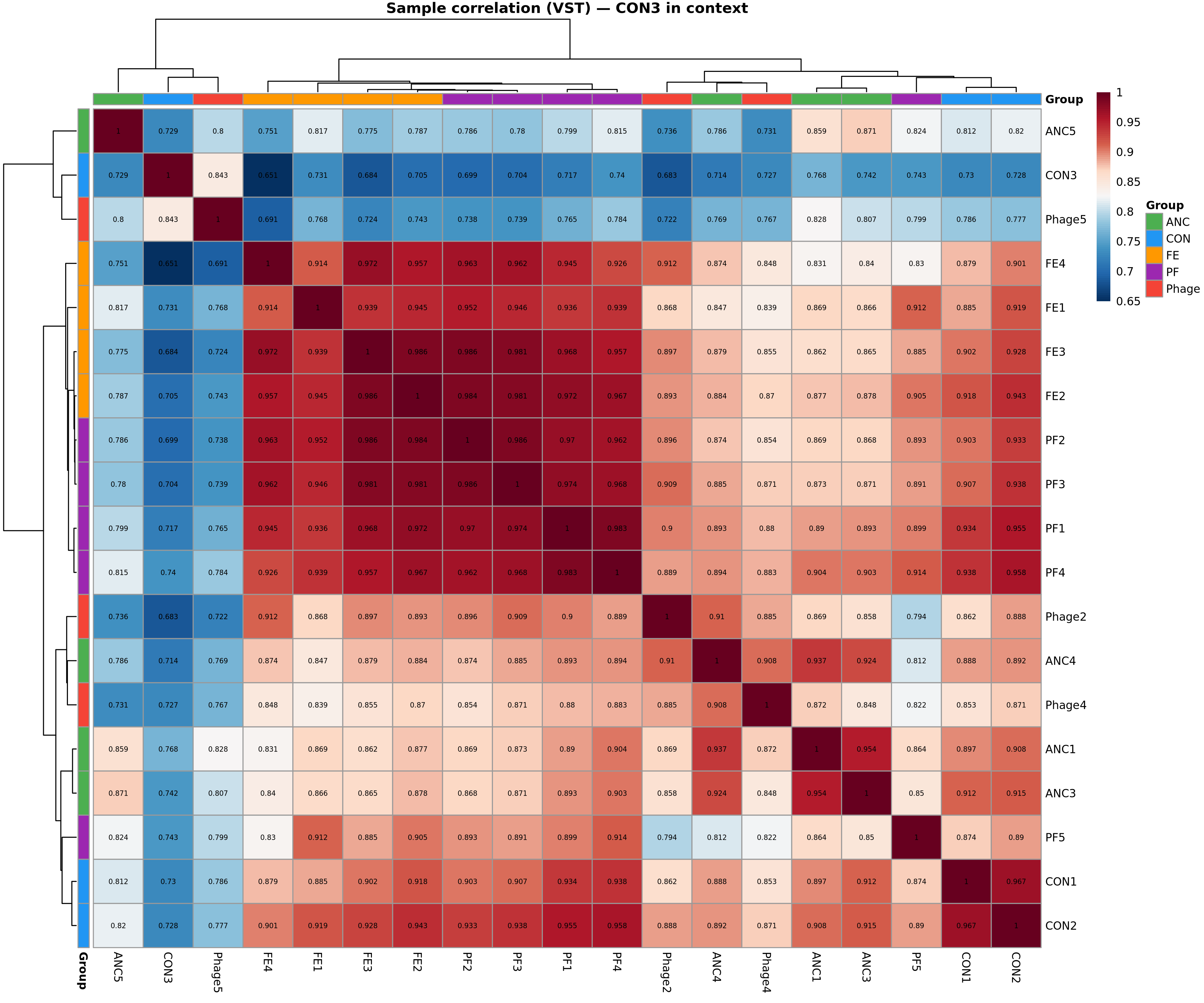

Supplement: Supplementary file 1 [file antibiotics-15-00684-s001.zip › SUPPLEMENTARY FOLDER/Sensitivity CON3/B_correlation_matrix.png]

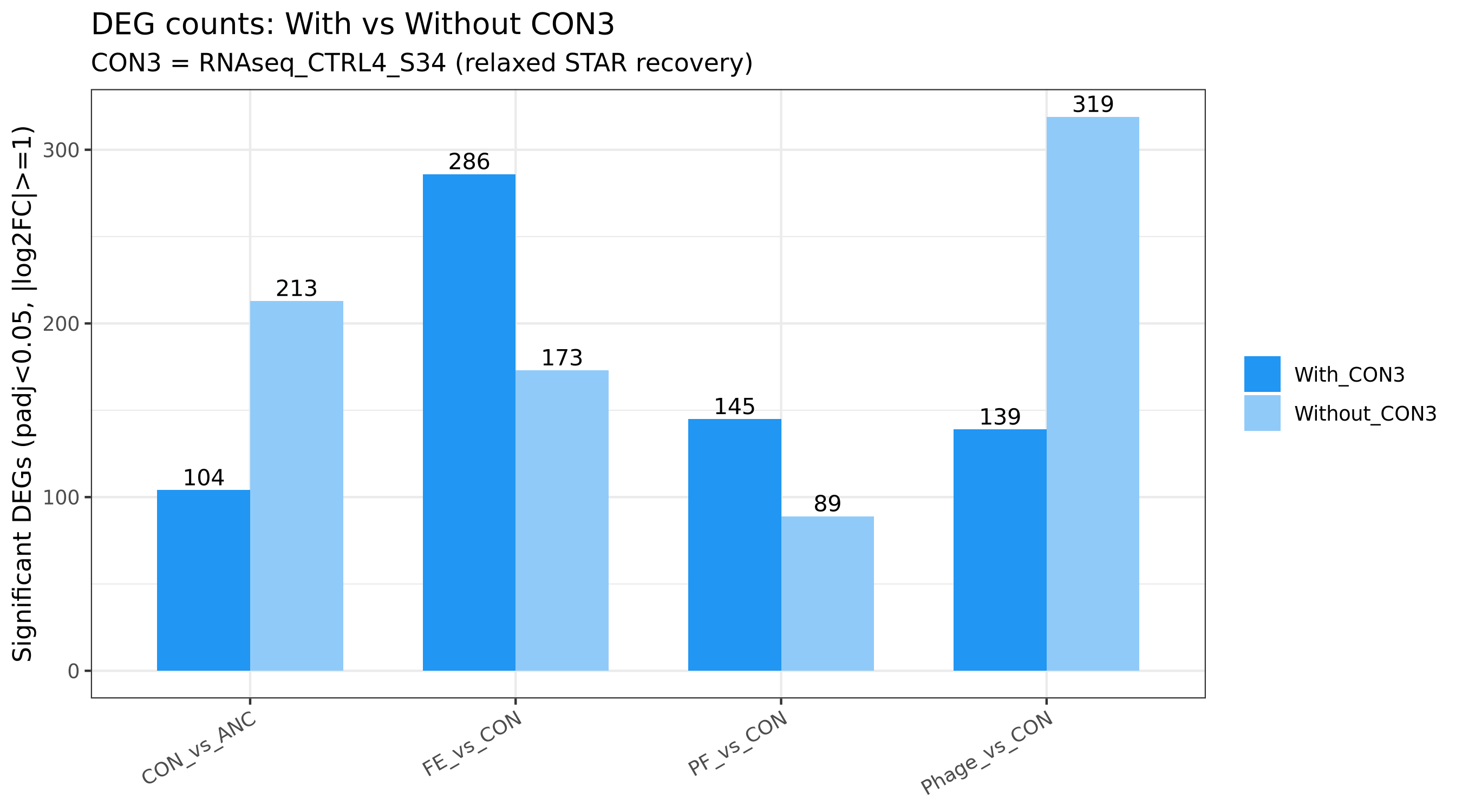

Supplement: Supplementary file 1 [file antibiotics-15-00684-s001.zip › SUPPLEMENTARY FOLDER/Sensitivity CON3/C_DEG_impact.png]

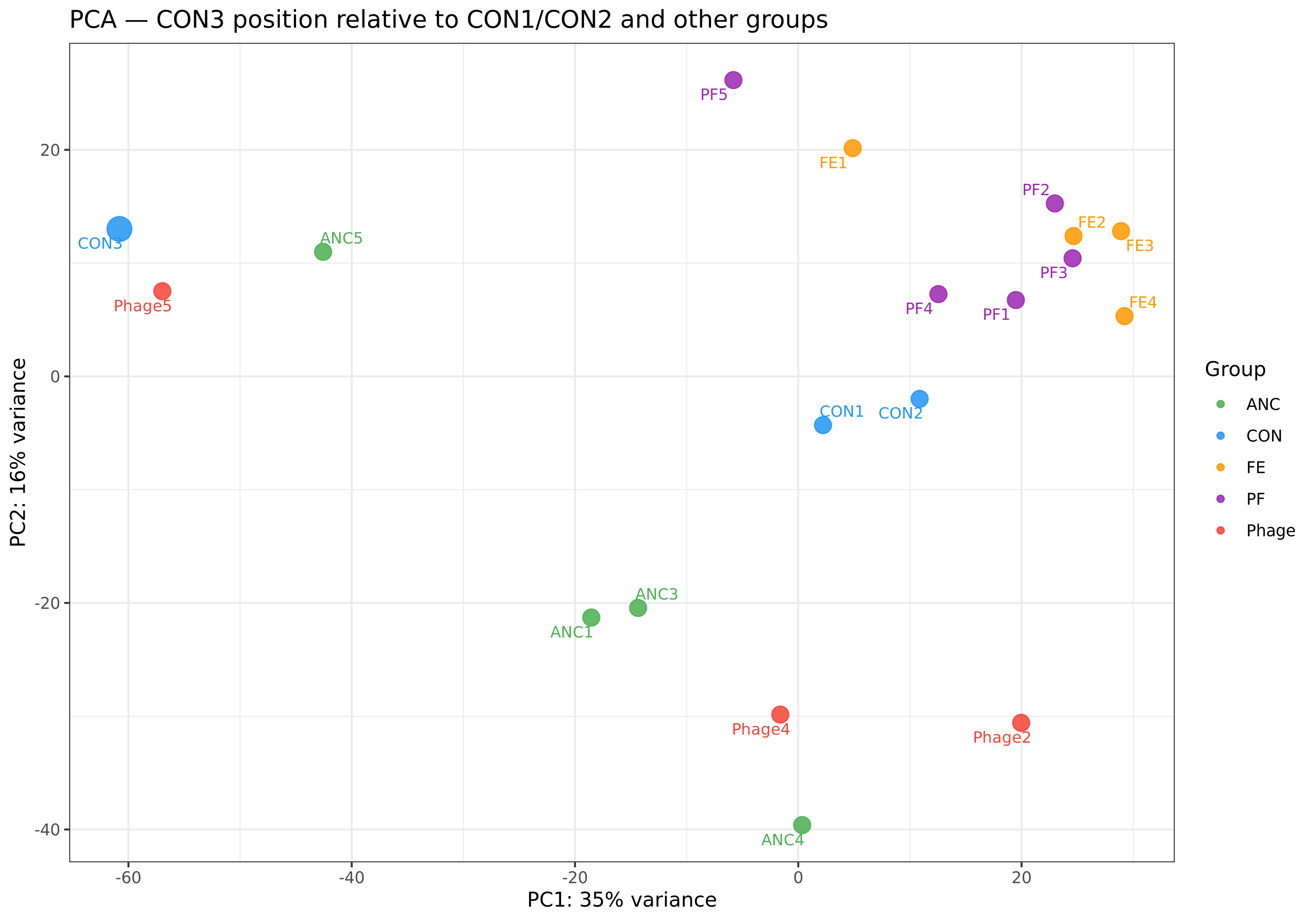

Supplement: Supplementary file 1 [file antibiotics-15-00684-s001.zip › SUPPLEMENTARY FOLDER/Sensitivity CON3/E_PCA_CON3_position.png]
